# Measurements of the size and correlations between ions using an electrolytic point contact

**DOI:** 10.1038/s41467-019-10265-2

**Published:** 2019-05-30

**Authors:** Eveline Rigo, Zhuxin Dong, Jae Hyun Park, Eamonn Kennedy, Mohammad Hokmabadi, Lisa Almonte-Garcia, Li Ding, Narayana Aluru, Gregory Timp

**Affiliations:** 1 Electrical Engineering and Biological Science, University of Notre Dame, Notre Dame, IN 46556 USA; 2 Department of Aerospace and Software Engineering and Research Center for Aircraft Parts Technology, Gyeongsang National University, Jinju, Gyeongnam 52828 Republic of Korea; 3 Mechanical Engineering, University of Illinois, Urbana, IL 61801 USA

**Keywords:** Nanoscale materials, Nanoscale devices, Nanopores, Nanometrology, Surfaces, interfaces and thin films

## Abstract

The size of an ion affects everything from the structure of water to life itself. In this report, to gauge their size, ions dissolved in water are forced electrically through a sub-nanometer-diameter pore spanning a thin membrane and the current is measured. The measurements reveal an ion-selective conductance that vanishes in pores <0.24 nm in diameter—the size of a water molecule—indicating that permeating ions have a grossly distorted hydration shell. Analysis of the current noise power spectral density exposes a threshold, below which the noise is independent of current, and beyond which it increases quadratically. This dependence proves that the spectral density, which is uncorrelated below threshold, becomes correlated above it. The onset of correlations for Li^+^, Mg^2+^, Na^*+*^ and *K*^*+*^-ions extrapolates to pore diameters of 0.13 ± 0.11 nm, 0.16 ± 0.11 nm, 0.22 ± 0.11 nm and 0.25 ± 0.11 nm, respectively—consonant with diameters at which the conductance vanishes and consistent with ions moving through the sub-nanopore with distorted hydration shells in a correlated way.

## Abstract (web summary)

The size of an ion affects everything from the structure of water to life itself. Here, a sub-nanometer diameter pore sputtered through a thin silicon nitride membrane is used to systematically test ion permeability by measuring the electrolytic current and current noise and show that the ions move with a grossly distorted hydration shell in a correlated way.

## Introduction

Chemistry, energy, even life itself depend on the size of ions dissolved in water held in a confined topography. It affects everything from the Helmholtz double-layer in a super-capacitor and blue-energy conversion efficiency to binding in a protein and the permeability of an ion channel^1–4^. It is no wonder then that the size has been the subject of penetrating scrutiny. The size of ions has been estimated empirically in a variety of ways, including x-ray and neutron scattering and double-difference infrared spectroscopy^5–7^, and theoretically using ab initio molecular dynamics (MD) simulations^1,8^. All of these studies seem to converge to a few specific conclusions: the gauge of an ion diameter—hydrated or not—is sub-nanometer, and the alkali ions (Na^+^, K^+^, and Cs^+^) are relatively weakly hydrated with a single shell of six to eight-coordinated water molecules, whereas Li^+^ and Mg^2+^ are more strongly hydrated with four to six coordinated waters and a second hydration shell^7,9^. The trouble with these measurements of ion size is that they were performed almost exclusively in concentrated electrolyte and more importantly, with few exceptions^2,10^, the measurements were not conducted in a well-defined, confined topography relevant to chemistry or biology. Yet, the properties of individual ions within a solution can only be defined at a hypothetical infinite dilution, where no ion–ion interactions occur, and importantly, confinement, especially below a nanometer, grossly affects the properties of water and ions^11–13^.

To remedy these deficiencies, in this report a sub-nanometer-diameter pore—i.e., sub-nanopore—ranging in diameter from 0.28 to 1.0 nm, sputtered through a thin (7–12 nm) silicon nitride membrane is exploited to systematically test ion permeability by measuring an electrolytic current through it^14–16^. Naively, ions in a viscous liquid are supposed to be impelled by an applied electric field to drift through the pore according to the electric force **F** = *z*_i_*e* · **E**, where *z*_*i*_ denotes the ion valence, *e* represents the elementary charge and **E** is the electric field, but retarded according to Stoke’s law, i.e., **F** = 3π*η·d*_i_·**v**, where *η* is the viscosity, *d*_*i*_ is the (hydrodynamic) ionic diameter and **v** is the velocity. When these forces balance, the ion reaches a drift velocity through the sub-nanopore, i.e., **v** = *μ*_i_·**E**, where *μ*_*i*_ = *ze*/3*πηd*_*i*_ is the ion mobility. Actually, in addition to the drifting motion, if there is a concentration gradient, diffusion (with a diffusivity of *D*_*i*_ = *k*_B_*Tμ*_*i*_/*e*, where *k*_B_ is Boltzmann’s constant and *T* is the absolute temperature) will also contribute to the current, and if there is a surface charge in the pore, electro-osmotic flow (EOF) has to be taken into account too. In particular, if there is a (negative) surface charge, the concentration of counter-(co-ions) in the pore can be higher(lower) than in the bulk, the transport becomes selective to cations, and the EOF in the electric field can affect the apparent mobility^17^.

When confined to a sub-nanometer scale, the viscosity of water is orders of magnitude larger than in bulk^18,19^. Thus, it was reasoned that the smaller the pore diameter became, the higher the viscosity and the lower the mobility until eventually ions would fail to permeate through the pore, which should be conspicuous in the conductance. Moreover, as the sub-nanopore shrinks relative to the ionic diameter, the electrolytic transport through it should become one-dimensional and the screening of the ion’s Coulombic potential by water should also diminish. Accordingly, the ionic motion in sub-nanopores should become highly correlated due to volume exclusion^20^ or Coulomb repulsion^21–23^. In a statistical analysis of the ionic motion, the conductance represents only the second moment of the current density, whereas noise represents the fourth moment^24^. So, it was reasoned that current noise would be a more sensitive gauge of the correlations between the ions than the conductance.

Here, it is shown that the electrolytic conductance through a sub-nanopore, which is mainly due to cations, vanishes when extrapolated to pores with a diameter smaller than 0.24 nm, which is about the size of a water molecule. This result indicates that ions permeate the pore with a grossly distorted hydration shell, which is consistent with MD simulations. Furthermore, a threshold is observed in the low frequency current noise power spectral density (PSD), below which the PSD is independent of current, and beyond which it increases quadratically with current. This dependence on current proves that the spectral density components of the noise, which are uncorrelated below threshold, are nearly perfectly correlated above it. Importantly, the onset of correlations in the noise current extrapolate to pore diameters: *d*_Li+_ = 0.13 ± 0.11 nm for Li^+^, *d*_Mg2+_ = 0.16 ± 0.11 nm for Mg^2+^, *d*_Na+_ = 0.22 ± 0.11 nm for Na^+^, and *d*_K+_ = 0.25 ± 0.11 nm for K^+^, which are consistent with the other estimates of de-hydrated ion sizes and consonant with the extrapolations derived from the conductance. Altogether, these data support the conclusion that, when it is forced through a sub-nanopore, the hydration shell of an ion is grossly distorted and the ionic motion is correlated at high current.

## Results

### Sub-nanopore fabrication and visualization

**Fig. 1:**
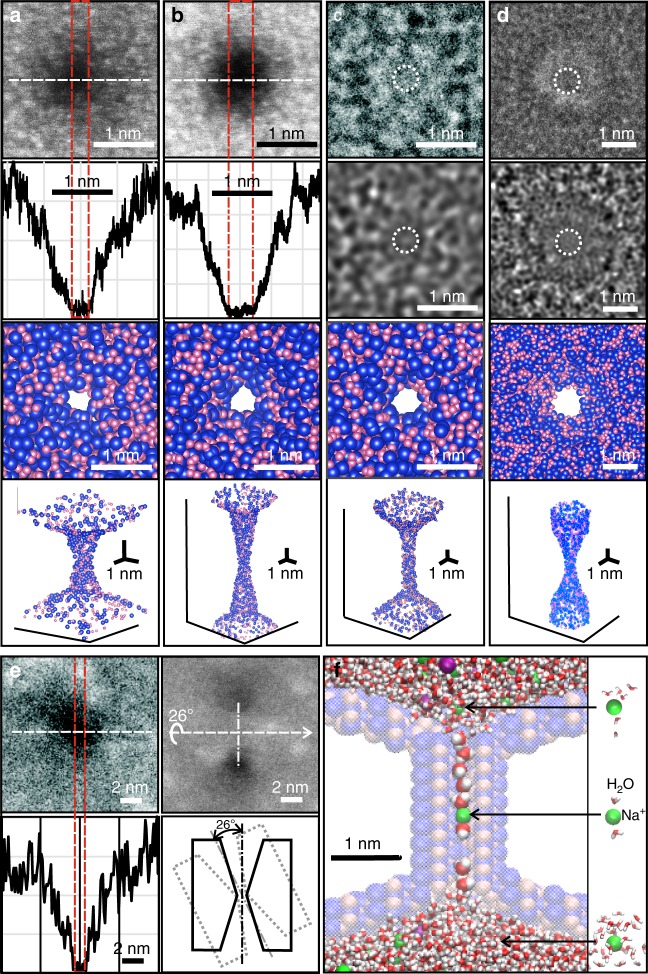
Sub-nanopores sputtered through silicon nitride membranes. (**a**, **b**; top) HAADF-STEM images of two sub-nanopores with mean-diameters at the waist of 0.28 nm and 0.40 nm, respectively, acquired with an aberration-corrected Themis Z. (**a**, **b**; 2nd from top) Line-plots associated with the white dashed lines in (a, b top). The shot-noise between the red dashed lines indicates the pore diameter. (**a**, **b**; 2nd from bottom) 2D-projections from the top through models of the sub-nanopores in (**a**, **b**; top) that indicate the atomic distribution near the pore waist. The atoms are depicted by space-filling models in which each *Si* is represented by a blue sphere and each *N* is a pink sphere. (**a**, **b**; bottom) 3D-perspectives of the space-filled models (**a**, **b**; 2nd from bottom). (**c**, **d**; top) TEM images of two sub-nanopores with mean-diameters at the waist of 0.42 nm and 0.70 nm, respectively, acquired with the Titan. The dashed circles delineate the shot noise that defines the pore waist. (**c**, **d**; 2nd from top) The corresponding multi-slice simulations of the TEM images (**c**, **d**; top) that were consistent with the imaging conditions. (**c**, **d**; 2nd from bottom) 2D-projections from the top through the models used in (**c**, **d**; 2nd from top) that indicate the atomic distribution near the pore waist. (**c**, **d**; bottom) 3D-perspectives of space-filled models of (**c**, **d**; 2nd from bottom). (**e**; top) HAADF-STEM images acquired from a pore with a mean-diameter of 0.89 nm at 0° (top, left) and 26° (top, right) tilt angles relative to the optic axis of the microscope. (**e**; bottom, left) The corresponding line-plot associated with the white dashed line (**e**; top) that indicates the mass-density under the beam. (**e**; bottom, right) An idealized model that illustrates the effect of the tilt angle on the image. **f** A snapshot extracted from MD of an idealized, negatively charged (−3*e*) 0.30 nm diameter sub-nanopore immersed in 1 M NaCl, illustrating the distortion of the hydration shell surrounding a Na^+^ (green) as it permeates through the (ghost) pore. The Cl^−^ (purple) are excluded from the pore because of de-hydration and electrostatic repulsion. Only the silicon (blue) and nitrogen (pink) atoms in the membrane at the pore surface are represented in the model, and even they are ghosted

A sub-nanopore spanning a silicon nitride membrane nominally 10 nm thick was created by sputtering with a tightly focused, high-energy electron beam in a scanning transmission electron microscope (STEM)^14–16^. Two different microscopes (an FEI Titan and aberration-corrected FEI Themis Z) were employed for sputtering and subsequently for visualizing the pore topography. Regardless of how the sub-nanopores were visualized, both the high-angle annular dark field (HAADF-)STEM (Fig. 1a, b, Supplementary Fig. 1) and TEM images (Fig. 1c, d, Supplementary Figs. 2, 3), combined with multi-slice simulations of them^25^, exposed the same features. The images revealed pores with a bi-conical topography, with cone-angles ranging from *θ* = 4–15° near the center of the membrane for the smallest pores, increasing to *θ* = 15−37° as the lumen opened, and an irregular waist with elliptical major and minor axes. Compelling evidence of the bi-conical topography was distilled from images acquired with HAADF-STEM under different tilt conditions relative to the axis of the electron beam. In HAADF-STEM, the image contrast develops from elastic scattering of electrons, which is a function of the atomic number Z and hence the mass. So, when the pore axis was tilted relative to the beam, both apertures of the pore were viewed simultaneously. The tilted images revealed sub-nanopores that were symmetric with apertures larger than the waist viewed at a zero tilt angle (Fig. 1e). Generally, the cross-sections at the waist, specified by the length of the minor/major axes, were estimated to range from 0.25 × 0.30 nm^2^ to 0.95 × 1.00 nm^2^.

As the mean distance between oxygen atoms in the water molecules within the first hydration shell surrounding a sodium ion was supposed to be about 0.24 nm^7^, the cross-section near the waist of these pores was determined to be less than some estimates of the completely hydrated ions^26–29^. This supposition was corroborated by MD simulations of the ion transport through an idealized sub-nanopore with a cylindrical waist and total negative surface charge of −3*e* distributed across the pore surface atoms (Methods). Snapshots taken from MD revealed a grossly distorted hydration shell around ions permeating through the sub-nanopore (Fig. 1f). Inside the pore, there were only two water molecules in the first hydration shell surrounding a Na^+^ counter-ion (green): one preceding and another following it through the pore, whereas in bulk electrolyte or in a pore >1 nm in diameter, the coordination number (defined as the number of oxygen atoms at a distance less than 0.25 nm from the ion) of Na^+^ and Cl^−^ is between 5 and 6. Thus, based on MD, even for the smallest diameters, ions were still hydrated inside the pore, but due to the confinement the number of water molecules in the hydration shell was lower than in the bulk.

The drastic change in the hydration shell in a sub-nanopore doubtless affected the ion permeability. This assertion followed from calculations of the potential mean force (PMF) on an ion in a sub-nanopore also accomplished with MD. The PMF (red lines in Supplementary Fig. 4) was estimated by integrating the mean force acting on an ion along the pore axis *z*, and then decomposed into ion-water (denoted as hydration, the blue line in Supplementary Fig. 4) and ion-pore components (denoted as electrostatic, the green line in Supplementary Fig. 4). Depending on the charge in the pore, the PMF barrier to Na^+^ ion permeation through a 0.30 nm diameter sub-nanopore was reduced to <35 *k*_B_*T* near the orifice (with a −3*e* surface charge) from 130 *k*_B_*T* (without charge). Moreover, due to the electrostatics, the PMF near the pore waist was so attractive that, once it entered, a cation was likely to remain there stably. Thus, the energy barrier against a cation permeating a negatively charged pore due to de-hydration was drastically reduced by the Coulombic attraction. On the other hand, Cl^−^ ions were both repelled by the negatively charged surface and de-hydrated by the confinement imposed by the sub-nanopore (PMF ~ 123 *k*_B_*T*), which blocked their permeation through the membrane and so, the transport through a sub-nanopore should be ion-selective.

### Electrolytic conductance through a sub-nanopore

**Fig. 2:**
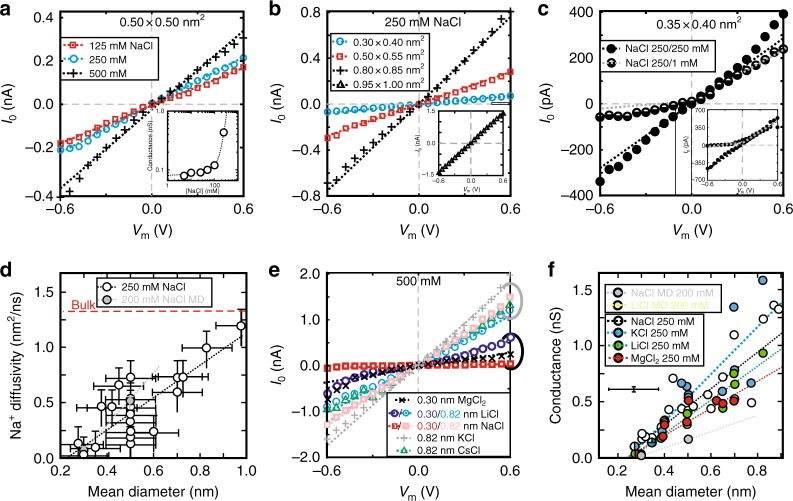
Electrolytic ion transport through sub-nanopores. **a** A juxtaposition of the current-voltage characteristics acquired from the same sub-nanopore with a 0.50 nm mean-diameter using different concentrations of NaCl electrolyte. The dotted lines reflect FESs. Inset: The conductance of a pore with a mean-diameter of 0.52 nm as a function of NaCl concentration, illustrating a minimum conductance for concentrations <100 mM. The dotted-line represents a match to the data assuming a surface charge of −0.19*e* nm^-2^. **b** Like **a**, but acquired from pores with 0.30 nm × 0.40 nm→)0.35 nm, (0.50 nm × 0.55 nm→)0.52 nm and (0.80 nm × 0.85 nm→)0.82 nm mean-diameters at the waist in 250 mM NaCl. Inset: Like **b**, but for a pore with a (0.95 nm × 1.00 nm→)0.97 nm mean-diameter. **c** The current–voltage characteristic of a sub-nanopore with a mean-diameter of (0.35 nm × 0.40 nm→)0.37 nm measured with 250 mM NaCl on both sides of the membrane (open circles), and then with a gradient across the membrane with only 1 mM NaCl on the *trans*-side (half-filled circles). The asymmetric conductance indicates that >97% of the current was carried by Na^+^. Inset: Like **c**, but for a pore with a mean-diameter of (0.75 nm × 0.85 nm→)0.80 nm. Here, 90% of the current was carried by Na^+^. **d** The dependences of the diffusivity extracted from FES on the mean-diameter of sub-nanopores for Na^+^ in 250 mM NaCl (open circles). The best-fit (black dotted) line extrapolates to zero diffusivity near a 0.22 nm-mean-diameter. For comparison, juxtaposed on the same plot is the diffusivity extracted from MD using model sub-nanopores with 0.30 nm and 0.50 nm diameters (gray circles). **e** Like **a**, but acquired from two sub-nanopores with (0.80 nm × 0.85 nm→) 0.82 nm (gray lasso) and 0.30 nm (black lasso) mean-diameters using different electrolytes at 500 mM. **f** The dependences of the conductance on the mean-diameter of sub-nanopores for four different electrolytes at 250 mM. The best-fit lines for the metal ions extrapolate to zero conductance at 0.21 ± 0.11 nm for Na^+^; 0.24 ± 0.11 nm for K^+^; 0.26 ± 0.11 nm for Li^+^ and 0.23 ± 0.11 nm for Mg^2+^, near to a 0.24 nm-diameter. For comparison, juxtaposed on the same plot are the conductances extracted from MD simulations performed using model sub-nanopores with 0.30 and 0.50 nm diameters in LiCl and NaCl. The error bars are typical of the standard deviation of the empirical data

To test the ion permeability, a sub-nanopore was first electro-wetted and then the voltage-dependence of the current through it was measured (see Methods section). Generally, when the electrolyte concentration was diluted (Fig. 2a) or the sub-nanopore diameter shrunk (Fig. 2b, Supplementary Fig. 5), the conductance (inferred from the slope at ±100 mV about 0 V) diminished. In concentrated electrolyte, ion–ion and ion–water interactions cause the actual number of available ions to be less than the number present, which is why the ion activity is used frequently as a gauge instead of concentration. At extreme concentrations (>1 M), the ions can be <1 nm apart on average, which interferes with the water network surrounding them and affects the number of counter-ions in the first solvation shell. In addition, at high concentration, ion-pairing develops that affects the spectral density of the individual carriers and therefore, the interpretation of the current and current noise distributions. So, to simplify the interpretation, even though data was acquired up to 2 M, most of the effort focused on concentrations ≤500 mM.

The conductance increased nearly linearly with the bulk electrolyte concentration when >100 mM. However, for dilute electrolyte concentrations <100 mM, a minimum conductance, *g*_min_, was routinely observed (Fig. 2a; inset). Earlier work indicated that bulk ions carry the current in concentrated electrolyte, whereas the conductance for dilute concentrations was attributed mainly to counter-ions compensating for the (negative) surface charge in the pore^14,17^. A rudimentary estimate of the surface charge density, *ρ*_s_ was obtained by measuring the conductance at different electrolyte concentrations spanning the range from 5 mM to 0.5 M and then extrapolating *g*_min_ to zero activity^17^. Phenomenologically, the minimum conductivity followed from: $\sigma _{\min } = 4\mu ^ + |\rho _s|/d$, where *d* denotes the pore diameter and μ^*+*^ the (cation) mobility, and so the resulting charge was estimated to range from *ρ*_s_ = −0.011 to −0.150*e* nm^−2^ for the sub-nanopores used in the work, which translated to as few as 3 or as many as 10 elementary negative charges on the surface. This was important because the surface charge affects (lowers) the PMF and facilitates cation permeability—according to MD, no cations surmount the energy barrier at the orifice of a charge-neutral pore (Supplementary Fig. 4). It also restricts the minimum effective dilution of the electrolyte to about 10−100 mM. Thus, an extrapolation to infinite dilution must start above this concentration.

If the pore surface charge was negative, then the conductance should be selective to cations^30,31^. This idea was tested by measuring the current through sub-nanopores with an electrolyte gradient (from 250 to 1 mM NaCl) imposed from the *cis*-side to the *trans*-side of the membrane (Fig. 2c). The current–voltage characteristics measured without a gradient (250 mM NaCl on both sides of the membrane) were more or less symmetric when the voltage was inverted, which was consistent with the (double-cone) topography of the sub-nanopores assuming a uniform surface charge distribution throughout. However, measurements with an electrolyte gradient imposed across the membrane revealed about a five-fold asymmetry in the current for a positive versus negative bias^31^. Three factors likely contributed to this asymmetry: first, the exclusion of Cl^−^ co-ions from the pore likely suppressed the anionic component to the current; second, the minimum conductance associated with the counter-ions must have affected the minimum current at negative bias; and finally, the additional series resistance due to the dilute concentration of electrolyte on the *trans*-side might have depressed the current, especially at high voltage.

To unravel how the surface charge, electrolyte diffusivity, concentration, and constituency in the pore contributed to the conductance, the distribution of the electric field, potential, current, and temperature were calculated using finite element simulations (FESs). The FESs leveraged an electro-hydrodynamic continuum model, specifically Poisson-Nernst-Planck (PNP) and Navier-Stokes (NS) theory, to describe the electrolytic transport. Generally, it has been found that the electrostatic potential, the electric field and the concentration of the counter-ions near a charged surface strongly depend on the excluded volume of the counter-ion, especially when the surface charge density becomes large, which would obviously affect the conductance and capacitance^32–34^. So, in this context, to improve the accuracy of the FESs, a modified Poisson-Boltzmann equation was used that accounted for the steric effects of the ions as well as the pore topography, drift, diffusion and EOF (see Methods section, Supplementary Tables 1, 2, and Supplementary Note #1). These simulations were additionally constrained by diffusivities and viscosities gleaned from the literature^18,19,35–37^. However, even this modified Poisson-Boltzmann approach suffered limitations. Specifically, it was not atomistic and did not include ion–water, water–water, and ion–ion correlations that were likely relevant to electrolytic transport. Yet, FESs like these have been used successfully to infer some of the essential aspects of electrolytic transport^32–34^.

From the matches between the FESs and the empirical current–voltage characteristics (Fig. 2a–c, e; dotted lines), it was inferred that the suppression of the conductance with negative bias voltage that occurred when a concentration gradient was imposed across the membrane was due to cations carrying >90.3 ± 3.1% of the current through sub-nanopores with a mean-diameter <0.83 nm (Fig. 2c; dotted lines). Thus, the negative surface charge likely squelched the Cl^−^ ion flux so that metal cations carried the current predominately. Parenthetically, according to the FESs, the electric field was focused near the waist of the sub-nanopore due to the bi-conical topography into a region less than 2 nm in extent depending on the cone-angle (Supplementary Fig. 6). The electric field was proportional to the current density, but despite the intense field near the waist, the current density there was small enough (*J* < 1 × 10^5^ A cm^−2^)to preclude Joule heating (Supplementary Fig. 6d and Supplementary Note #1)^38^.

To account for the empirical data acquired at low voltage without a concentration gradient, the diffusivities inferred from FESs monotonically collapsed to zero near a sub-nanopore mean-diameter of about 0.22 ± 0.11 nm (Fig. 2d, open circles, Supplementary Fig. 7). A linear extrapolation was justified because the diffusivity was supposed to depend inversely on the viscosity, which in turn was supposed to depend inversely on the diameter^18,19^. For example, the Na^+^ diffusivities, *D*_Na+_, inferred from FESs ranged from *D*_Na+_ = 0.03 nm^2^ ns^−1^ to 1.19 nm^2^ ns^−1^, which were smaller than the corresponding bulk value (*D*_Na+_ = 1.33 nm^2^ ns^−1^). The diffusivity inferred this way was validated by MD using pores with diameters of 0.30 and 0.50 nm, which likewise indicated that the diffusivity collapsed as the diameter shrunk, extrapolating to zero at a diameter of 0.27 nm (Fig. 2d, gray circles). A similar trend has also been observed in prior MD studies of the dynamics of Na^+^ in model (proteinaceous) ion channels^37^. Both of these studies tracked with an increase in the viscosity of water confined on a nanometer-scale^35^.

Interestingly, the specific metal cation (whether hydrated or not) also subtly affected the conductance depending on pore diameter. This effect was apparent in the dispersion of the conductance between different electrolytes. For example, as the sub-nanopore (geometric) mean-diameter shrunk, the observed relative standard deviation (RSD) in the conductance grew (Fig. 2e, f, Supplementary Figs. 5, 8 and note #2), which was attributed to the difference between the sizes of the hydrated and de-hydrated ions carrying the current (Supplementary Table 3). Likewise, depending on the concentration, the conductance could be selective to the type of ion. For example, according to other work^8,30^, even though the de-hydrated K^+^-cation (0.298 nm) was supposed to be larger, it was more weakly hydrated than Na^+^ (0.234 nm), which allowed for greater distortion of the hydration shell, and so the larger ion could permeate a smaller pore more easily compared to the smaller hydrated Na^+^. Correspondingly, the K^+^ conductance was observed (sometimes) to be larger than the Na^+^ conductance in the same sub-nanopore, i.e., $g_{{\mathrm{K}}^ + } > g_{{\mathrm{Na}}^ + }$. Consistent with this premise, since Li^+^ and Mg^2+^ were supposed to be more strongly hydrated^7,9^ with de-hydrated diameters such that *K*^+^(0.298 nm) > Li^+^(0.188 nm) > Mg^2+^(0.144 nm), it makes sense then that $g_{{\mathrm{K}}^ + } > g_{{\mathrm{Li}}^ + } > g_{{\mathrm{Mg}}^{2 + }},$ as was often observed.

Tellingly, regardless of the electrolyte constituency (NaCl, KCl, LiCl, or MgCl_2_) or activity, as the pore diameter shrunk, the conductance collapsed (Fig. 2f, Supplementary Fig. 8). Because the membrane was so thin and the electric field distribution about the waist so narrow and the diameter so small, it was conjectured that a sub-nanopore acted essentially like an electrolytic point contact^39,40^. Since the conductance through an ideal point contact associated with a circular hole of diameter *d* through a vanishingly thin membrane, immersed in electrolyte of conductivity *σ*, scaled linearly with the diameter according to *g* = *σ* ⋅ *d*, a linear extrapolation to zero conductance was used as a measure of the size of the metal ions. The best-fit lines extrapolated to zero conductance at 0.21 ± 0.11 nm for Na^+^; 0.24 ± 0.11 nm for K^+^; 0.26 ± 0.11 nm for Li^+^ and 0.23 ± 0.11 nm for Mg^2+^, which were in-line with other estimates for the (de-hydrated) ion diameters (Supplementary Table 3). The statistic *R*^2^, which tells how close the data were fitted to a regression line, was *R*^2^ = 0.92, 0.86, 1.0, and 0.85 for NaCl, KCl, LiCl, and MgCl_2_, respectively, over the range of diameters <0.5 nm. (*R*^2^ = 1.0 indicates that the model explained all the variability of the data.) The average of the intercepts for all the metal ions recovered from a linear extrapolation assuming an ideal point contact, i.e., *d*_M_ = 0.24 ± 0.11 nm, was reproduced even assuming a thick membrane with a power-law governing the dependence of the conductance on diameter (Supplementary Note #3). Finally, MD simulations of the conductance performed sparingly for NaCl and LiCl electrolyte in idealized pores with 0.30 and 0.50 nm diameters validated this same trend also, extrapolating to zero at a diameter of 0.27 nm. Coincidently, these intercepts were all about the size of a water molecule (0.28 nm)^41^, which lends support to the idea that the hydration shell was grossly distorted.

Due to its small size, it was speculated that a proton would permeate through a sub-nanopore below the threshold for metal ion conductance via a Grotthaus-like mechanism by which it hops or tunnels through the hydrogen bond network of water molecules^42^. Regardless of the metal cation, the electrolyte solutions were all weakly acidic near pH 6 (see Methods section, Supplementary Table 4), but since the proton concentration at pH 6 was miniscule in comparison to the metal ions ([H^+^] = 1 μM), the conductance generally vanished in the smallest sub-nanopores below 0.24 nm (Fig. 2f, Supplementary Fig. 8). On the other hand, it was reasoned that, in the absence of other electrolytes, at a lower pH the proton concentration would increase and the conductance along with it, even if the pore diameter was smaller than a water molecule^42^. To test this idea, concentrated HCl was introduced into the pore to decrease the pH to 1 ([H^*+*^] = 100 mM). HCl was chosen because it supposedly does not etch silicon nitride with a low oxide content^43^. Unlike the current measured in electrolyte at pH 6 (Supplementary Fig. 9b; red traces), the current traces acquired in concentrated HCl fluctuated erratically at high voltage (Supplementary Fig. 9a, b; blue traces)^44–46^. Typically, the time-averaged conductance increased in concentrated HCl regardless of the pore diameter (Supplementary Fig. 9a), which could be attributed to the increased diffusivity of protons over metal cations or excess protons tunneling across a hydrophobic void in a sub-nanopore, but only equivocally (Supplementary Note #4).

Based on the extraploated size of the pore at which the conductance vanished (Fig. 2f), the dispersion in conductance measured by the differences in the RSD between large and small pore diameters, and MD simulations of the ion transport, it was inferred that hydrated ions likely permeated the larger diameter pores, whereas a cation with a grossly distorted hydration shell mainly carried the current through the smaller ones. The point contact was supposed to introduce a barrier to permeation because of the energy required to de-solvate the ions^47,48^, but that barrier diminished as the negative surface charge in the sub-nanopore increased, and so it was likely that the hydration layer would peel off as a cation was impelled through the sub-nanopore.

**Fig. 3:**
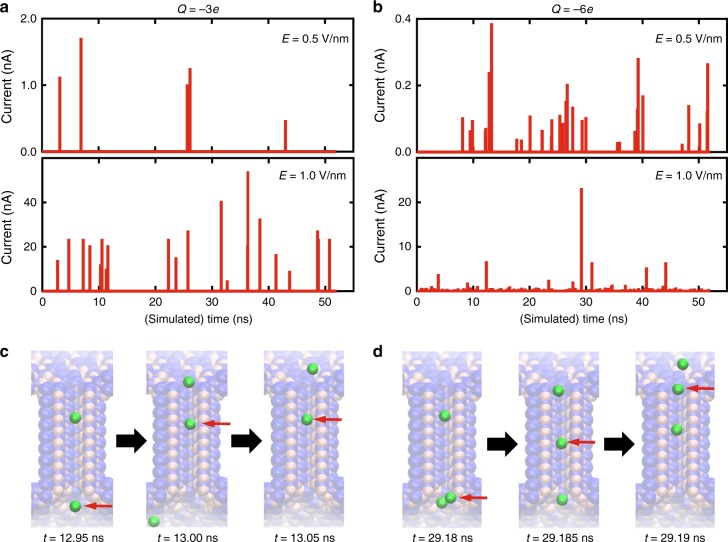
Visualization of the current through an idealized sub-nanopore accomplished with molecular dynamics (MD). **a** MD simulations of ionic currents traces through a 0.30 nm-diameter sub-nanopore with the total charge of *Q* = −3.0*e* in 200 mM NaCl electrolyte are shown for two different electric fields: *E* = 0.5 and 1.0 V nm^−1^. The current spikes represent Na^+^ cations translocating across the membrane through the sub-nanopore, and the magnitude of spikes is related to the duration of the translocation. **b** Like **a**, but with a total charge of *Q* = −6.0*e*. With increasingly negative *Q*, translocations are observed more frequently and the magnitude of the current spikes increases in the higher electric field. **c** Snapshots extracted from the MD simulation are shown illustrating the trajectories of ions through the idealized sub-nanopore at *E* = 0.5 V nm^−1^. At this low field, the ion trajectory was governed essentially by the electric field and single ions approach the orifice one-at-a-time. The red arrows track the same ion. **d** Like **c**, but for a larger electric field (1 V nm^−1^). Now, the ion traffic near the orifice becomes congested with one ion accelerating the translocation of another through the pore via electrostatic repulsion. Thus, the ion motion becomes correlated

MD offered a penetrating, atomistic perspective of the ion conductance through a sub-nanopore, but it also suffered limitations. In particular, MD was computationally demanding, and economical simulations of the conductance generally proved to be incommensurate with the limited bandwidth and/or low electric fields and the narrow electric field distribution^40,49^ characteristic of the measurements (Supplementary Figs. 10, 11). Nevertheless, it was still possible to glean insight by using MD to inform on the current by tracking individual cations through a sub-nanopore. It was discovered that when Na^+^ ions were electrically impelled through a 0.30 nm-diameter sub-nanopore, spikes appeared in the current traces (Fig. 3a, b). Unlike pores with a large diameter (≥1 nm) where the ion flow was practically continuous (Supplementary Fig. 10), the current spikes in a sub-nanopore were incontrovertibly due to counter-ions transiting rapidly through the pore with an amplitude related to the dwell time. With increasingly negative surface charge (*Q* = −3.0*e* → −6.0*e*), the translocations occurred more frequently because the energy barrier was lower so the cation permeated into the pore more easily, and with increasing field the amplitude of the spikes improved due to the acceleration of the ion. Generally, ions diffused up to and were eventually captured by the electric field extending only a few nanometers above the orifice of the pore. For the lower field, counter-ions approached the cylindrical waist of the pore one-at-a-time governed mainly by the field (Fig. 3c, red arrows). However, for the larger field, more than one ion impinged on the orifice at the same time (Fig. 3d, red arrows) so that the traffic through the pore became congested and was affected, not only by the field, but by Coulombic repulsion between ions as well.

### Current noise in a sub-nanopore

It was reckoned that correlated ion transport would not be easily observed in the conductance, however, due to the limited bandwidth of the measurements. So instead, since it has already been established that *1/f* noise informs on local current fluctuations^24^, noise measurements were used to improve the sensitivity to correlations.

**Fig. 4:**
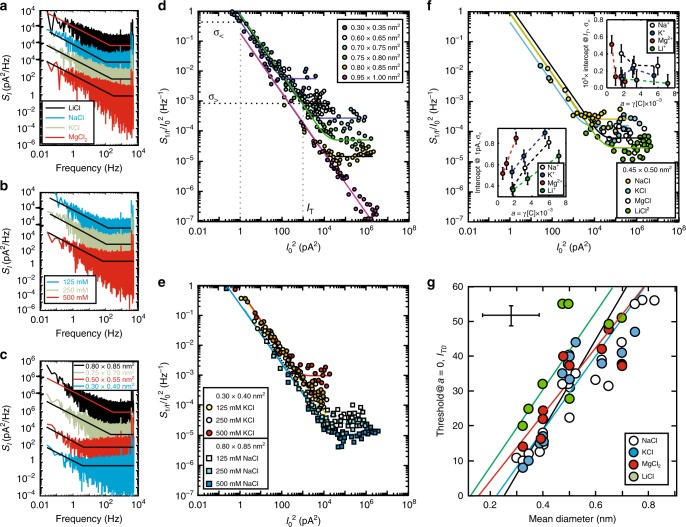
Correlated current noise in sub-nanopores. **a** The noise power spectral densities (PSDs) acquired from a sub-nanopore with a mean-diameter of 0.72 nm at the waist, at about the same pore current (*I*_0_ = 370–465 pA) for four different electrolytes at 250 mM. The spectra are offset for clarity. **b** Like **a**, the PSDs acquired from the same sub-nanopore at about the same open pore current (*I*_0_ = 410–440 pA) for three different concentrations of NaCl. **c** Like **a**, the PSDs acquired from four different pores with mean-diameters of (0.30 nm × 0.40 nm→)0.35 nm, (0.50 nm × 0.55 nm→)0.52 nm, (0.70 nm × 0.75 nm→)0.72 nm, and (0.80 nm × 0.85 nm→)0.82 nm, measured in 500 mM KCl. **d** The noise PSD intercept at 1 Hz (*S*_*1/f*_), normalized by the square of the open pore current, $$I_0^2$$, measured in 500 mM NaCl, plotted as a function of $$I_0^2$$ for sub-nanopores with mean-diameters ranging from (0.30 nm × 0.35 nm→)0.32 nm to (0.95 nm × 1.00 nm→)0.97 nm. The normalized noise power for *I*_0_ < 10 pA was generally consistent with noise resulting from uncorrelated current fluctuations. However, beyond the threshold current defined by *I*_T_, for the 0.288 nm-diameter pore, $$S_I/I_0^2$$ is independent of the current indicating correlations between fluctuations. The solid lines represent fits to the data. The (black) dotted lines represent the extrapolations from the fits to the intercepts σ_<_ and σ_>_ at which $$S_I/I_0^2$$ reaches 1 pA (below threshold, left vertical gray dotted line) and $$S_I/I_0^2$$$$I_0^2$$ at threshold (right vertical gray dotted line). **e** Like **d**, but for two different electrolytes at three concentrations, using respectively two different pores: one with a (0.30 nm × 0.40 nm→)0.35 nm (top) and another with a (0.80 nm × 0.85 nm→)0.82 nm mean-diameter (bottom). **f** Like **d**, but for four different electrolytes at 500 mM concentration for a sub-nanopore with a (0.45 nm × 0.50 nm→)0.47 nm mean-diameter. **g** The dependence of the threshold current extrapolated to zero-activity, *I*_T0_, on the mean-diameter of the pore is shown for four different electrolytes. The best-fit lines extrapolate to diameters for Li^+^, Mg^2+^, Na^+^, and K^+^ of 0.13 ± 0.11 nm, 0.16 ± 0.11 nm, 0.22 ± 0.11 nm, and 0.25 ± 0.11 nm, respectively, consistent with estimates derived from the conductance. The error bars represent the standard deviation

Current noise was inescapable (Fig. 4a–c) and correlations in it were conspicuous (Fig. 4d–f). When a voltage bias was applied to a sub-nanopore immersed in electrolyte, regardless of the electrolyte constituency, the activity or pore diameter, the low frequency current noise PSD had at least two components to it: a (pink) 1/*f*-component and an excess, frequency-independent (white) noise component between 100 Hz and 10 kHz (Fig. 4a–c)^50–52^. The noise spectra were classified over the entire frequency range by fitting to: $S_I = S_{1/f}\frac{1}{f} + S_0 + S_1f + \cdots$, to extract the parameters, *S*_*1/f*_, *S*_0_, and *S*_1_, which were then used to gauge, the amplitude of the 1/*f*, white and dielectric noise, respectively. With the exception of the data acquired in concentrated *HCl*, the noise between 0.1 < *f* < 100 Hz was observed to be inversely proportional to the frequency, i.e., *S*_1/*f*_ ~ *f*^−*β*^, but it was not universally so that *β* = 1, but rather 0.8 < *β* < 1.3, increasing for larger current. On the other hand, when the exponent was forced to fit *β* = 1, the amplitudes *S*_1*/f*_ and *S*_0_, were both found to be independent of the current for *I*_0_ ≤ 1 pA with an abrupt increase above a threshold, *I*_T_, that depended on the pore diameter, the constituency and concentration of electrolyte (Fig. 4d–f).

The 1*/f* noise for *I*_0_ < 10 pA was independent of the current since the normalized current noise followed $S_I/I^2\sim 1/I_0^2$, regardless of the pore cross-section at the waist (Fig. 4d), the concentration (Fig. 4e) or electrolyte constituency (Fig. 4f). These observations regarding 1/*f* noise were consistent with Weissman’s prediction that *S*_*I*_ → *V*/(*π*^2^*d*^3^)(1/*f*) for a point contact with diameter *d*, where *V* was the scaling volume that depended on the source of the fluctuations^53^. However, Weissman’s model was probably too simplistic to account for noise in a sub-nanopore because, it ignored the size of an ion relative to the diameter and fluctuations in nanofluidic transport^22^, and it produced an electric field that was not an analytical function.

The noise power measured at low current (*I*_0_ ≤ 10 pA) was attributed to the uncorrelated motion of metal ions in the sub-nanopore. To see why, the PSD was related to the current autocorrelation function through a generalization of the Wiener-Khinchin theorem^54^, i.e., *C* = 〈Δ*I*(*t*)Δ*I*(*t* + *δt*)〉, where Δ*I* = *I*(*t*) − 〈*I*〉 is the noise current and 〈*I*〉 is the average current. To illuminate the correlations in the noise power, it was normalized so that: *S*_*I*_/*I*^2^ = 〈Δ*I*^2^〉/〈*I*〉^2^ Γ(*f*/f*), where f* denotes a relaxation time^52^. If the average current is given by: 〈*I*〉 = *Ni*, where *N* measures the number of carriers and *i* is the current carried by a single carrier, then the variance of the sum of the single particle currents must be the sum of their covariances: i.e., ${\mathrm{Var}}\left( {\mathop {\sum}\limits_{n = 1}^N {i_n} } \right) = \mathop {\sum}\limits_{n = 1}^N {\mathop {\sum}\limits_{m = 1}^N {{\mathrm{Cov}}(} } i_n,i_m) = \left\langle {{\mathrm{\Delta }}i^2} \right\rangle \left[ {(N) + N(N - 1)\xi } \right],$ where *ξ* represents the average correlation between the single particle spectral densities. If the single particle currents were uncorrelated, then *ξ* = 0 so that $S_I/I^2\sim \left\langle {{\mathrm{\Delta }}I^2} \right\rangle /\left\langle I \right\rangle ^2 = N\left\langle {{\mathrm{\Delta }}i^2} \right\rangle /N^2i^2\sim N/I_0^2,$ which accounted for the observation that the normalized noise power scaled like the inverse square of the current for *I*_0_ ≤ 10 pA and linearly with the activity, regardless of the cation (Fig. 4f; lower left inset). The dependence on activity was inferred from *σ*_<_, which is an extrapolation of $S_{1/f}/I_0^2$ to *I*_0_ = 1 pA (see Methods section and the definitions in Fig. 4d).

On the other hand, as the current increased above about *I*_0_ > 10 pA, generally a threshold, *I*_T_, was observed beyond which the normalized noise power remained relatively independent of the current such that: $S_{1/f}/I_0^2\sim 1$ (Fig. 4d–f). The threshold current was defined by the intersection of the normalized power $S_{1/f}/I_0^2\sim 1/I_0^2$ with the deviation $\left( {S_{1/f}/I_0^2\sim 1} \right)$ from it (see Methods section). It was asserted that the threshold signaled the onset of correlations since, for statistically independent carriers, when the average correlation between single particle spectral densities approaches *ξ* → 1, the normalized noise power should be relatively independent of the current, i.e., *S*_*I*_/*I*^2^ ~ 〈Δ*I*^2^〉/〈*I*〉^2^ = 〈Δ*i*^2^〉[(*N*) + *N*(*N* − 1)*ξ*]/*N*^2^*i*^2^ = 〈Δ*i*^2^〉/*i*^2^ ~ 1. Not only that, but also σ_>_, inferred from $S_{1/f}/I_0^2$ at *I*_T_ (see Methods and definitions in Fig. 4d), was relatively independent of the activity (except for Mg^2+^, Fig. 4f; upper right inset), which further supported the assertion that the ion motion was correlated.

To rigorously test the idea that the noise current was correlated, the dependence on pore diameter, the electrolyte constituency and concentration were all measured. It was reasoned that shrinking the pore diameter relative to the ionic diameter would improve correlations due to the steric constraint and reduced dielectric permittivity, and so reduce the threshold current. Furthermore, it was argued that diluting the electrolyte concentration would boost the correlation coefficient because the number of mobile cations in the pore would be reduced (as per Fig. 3), thereby improving the prospects for pair-wise coordination. Invariably, it was observed that the noise threshold current decreased as the pore diameter shrunk or when the electrolyte activity diminished or when the size of the ion grew larger relative to the pore diameter (Fig. 4d–g), validating the hypothesis that the threshold was due to correlated ion motion.

Importantly, after inferring the zero-activity (infinite dilution) threshold current, *I*_T0_, from the concentration-dependence of *I*_T_ for each pore (Supplementary Figs. 12, 13), linear extrapolations of the pore diameters to *I*_T0_ = 0 (Fig. 4g) indicated cation sizes that practically coincided with those derived from the conductance (Fig. 2f) and prior (de-hydrated) estimates (Supplementary Table 3). The linear extrapolation was justified since the threshold current, *I*_T_, defined by the value at the cross-over to correlated motion: i.e., $S_I/I^2\sim \left\langle {\Delta I^2} \right\rangle /\left\langle I \right\rangle ^2 = N\left\langle {\Delta i^2} \right\rangle /N^2i^2\sim N/I_0^2 \to 1$, was supposed to depend on the square root of the number of carriers in the pore, which scaled like the diameter. The linear extrapolation of *I*_T0_ to zero (solid lines in Fig. 4g) indicated a minimum pore diameter of *d*_Li+_ = 0.13 ± 0.11 nm for Li^+^, which was comparable to estimates of the de-hydrated diameter, but much smaller than estimates of the hydrated diameter (Supplementary Table 3). Similarly, the diameters for Mg^2+^, Na^+^, and K^+^ were estimated to be *d*_Mg2+_ = 0.16 ± 0.11 nm, *d*_Na+_ = 0.22 ± 0.11 nm, and *d*_K+_ = 0.25 ± 0.11 nm, respectively, all comparable to estimates of de-hydrated ions (*d*_Mg2+_ = 0.144 nm, *d*_Na+_ = 0.234 nm, and *d*_K+_ = 0.298 nm) and much smaller than the hydrated diameters. The near-coincidence between the cation sizes derived from the extrapolations of the conductance and the noise threshold supports the idea that they measured the same thing, but the noise measurements offered superior discrimination since Li^+^ and Mg^2+^ were gauged to be smaller than Na^+^ and K^+^. Thus, the threshold current ascribed to correlations in the ionic motion was used to infer the size of de-hydrate cations.

Doubtless volume exclusion, ion–ion interactions and interactions between ions and the water network^20,55,56^, especially at high electrolyte concentration and small pore diameter, affected the estimate of *I*_T_ used to determine the cation size. However, contrary to the notion that concentrating the electrolyte induces correlations^23^, the data indicated otherwise. Instead, the current threshold, *I*_T_, observed in the noise increased with activity, which was interpreted to mean that higher concentration frustrated the onset of correlations. To punctuate this argument, additional measurements were performed up to 2 M concentration using various electrolytes, including NaCl, in sub-nanopores, ranging from a mean diameter of 0.32 to 0.6 nm (Supplementary Fig. 13). With increased activity, the threshold current *I*_T_ was repeatedly observed to increase in the range of currents explored here. Moreover, consistent with the trends inferred from more dilute concentrations, linear extrapolations of the data to zero activity indicated about the same threshold *I*_T0_ with *R*^2^ = 0.992. Thus, it was inferred that correlations in the ion motion were actually degraded by increasing the electrolyte concentration.

From evidence like that in Fig. 4 (and Supplementary Figs. 12, 13) and the MD in Fig. 3, it was argued that the dependence of the threshold on activity and pore topography was probative, indicating how the number of ions in the pore volume affected the correlations. Usually a reduction in the threshold was observed as the electrolyte was diluted (Fig. 4f; insets, Supplementary Figs. 12, 13) or as the pore diameter was reduced relative to the (de-hydrated) ionic diameter (Fig. 4g), but not always. Curiously, no threshold was evident within the current range *I*_0_ > 10–1000 pA for a 0.35 nm-mean-diameter pore in 125 mM KCl (Fig. 4e) and likewise for a 0.97 nm-mean-diameter pore immersed in 500 mM NaCl (Fig. 4d), although for the latter case the threshold may have just exceeded the current range tested.

From this evidence, bounds on the minimum and maximum number of correlated ions were estimated from the pore volume and electrolyte concentration. For example, for a pore with a 0.35 nm-diameter with a cone-angle of 5° in a membrane 10 nm thick, filled with 125 mM KCl, it was estimated that less than one (0.4) *K*^+^ was in the pore volume of 5.4 nm^3^. Likewise, the pore with a 0.97 nm-diameter and a cone-angle of 20° immersed in the 500 mM NaCl electrolyte should contain about twenty-one Na^+^ in a volume of 69.8 nm^3^. Thus, the lack of a threshold indicated that correlations could be disrupted in two ways; either by using a pore that was: (1) too small in dilute electrolyte, such that the volume contained too few (<1) ions; or (2) too large in concentrated electrolyte, such that the volume contained too many (>20) ions to observe a threshold within the current range. So, it was inferred that a reduction in the pore volume relative to the size of the ion or its activity could destroy correlations resulting in a reduction in the 1/*f* noise above the threshold current. This inference was corroborated by the improved signal-to-noise ratio observed in the blockade current associated with the translocation of protein homopolymers through a sub-nanopore^16^. The concomitant reduction in the unoccluded volume through the introduction of a protein into the sub-nanopore was apparently enough to disrupt correlated ion motion (Supplementary Fig. 14).

## Methods

### Sub-nanopore fabrication and visualization

Pores with sub-nanometer cross-sections were sputtered through thin, custom-made silicon nitride membranes (SiMPore, Inc. West Henrietta, NY) using a tightly focused, high-energy (300 kV) electron beam carrying a current ranging from 300–800pA (post-alignment) in a scanning transmission electron microscope (STEM, FEI Titan 80-300 or FEI Themis Z, Hillsboro, OR) with a field emission gun (FEG)^15,16^. The silicon nitride film that formed the membrane was deposited by LPCVD directly on the top surface of a polished silicon handle wafer and the membrane was revealed after lithography using an EDP (an aqueous solution of ethylene diamine and pyrocatechol) chemical etch through a window on the polished back-side of the handle wafer. The thickness of the membranes, which ranged from *t* = 7.8 to 12.3 nm, was measured in situ using electron energy loss spectroscopy (EELS) or on a sister chip from the same lot just prior to sputtering a pore. The roughness of the membrane, measured with custom-built silicon cantilevers (Bruker, Fremont, CA) with 2 nm radius tips, was estimated to be <0.5 nm-rms, typically, but increased as the membrane became thinner.

After sputtering, the sub-nanopores were visualized in situ or re-acquired with either high resolution transmission electron microscopy (HRTEM) or high-angle annular dark field (HAADF-)STEM. To minimize beam damage, the sub-nanopores were examined using low beam current (<10–30 pA) or low energy (80 kV) or both (Supplementary Fig. 1). The illumination convergence angle in the Titan was typically *α* = 10 mrad at 300 kV, whereas in the Themis Z, *α* = 18 mrad at 300 kV or *α* = 27.1 mrad at 80 kV with a monochromator limiting the energy dispersion in the range 200–220 mV at 80 kV according to EELS.

HRTEM and HAADF-STEM are powerful tools for structural analysis; no other tools boast higher resolution. Generally with TEM, there are essentially two strategies that inform on the topography, which can be differentiated aberrations. The first involves the reconstruction of the exit-plane wave using a series of images acquired at different focus settings and or tilt angles, which amounts to a holographic method that recapitulates the phase information lost in forming an (intensity) image of the wave function. The second involves using aberration-corrected lenses to extend the point resolution. We used both.

The point resolution of the TEM corresponds to the extended Scherzer de-focus, where the contrast transfer function (CTF) of the microscope first crosses the spatial frequency (*k*) axis. Whereas the CTF is zero at the origin, it becomes positive for intermediate values of *k*. In this region of *k*, all structural information was transferred with positive phase contrast, i.e., the scattering centers (atom positions) appear with dark contrast. Therefore, the information in HRTEM images was directly interpretable up to the point resolution. The point resolution can be expressed as: $r_{{\mathrm{sch}}} = 0.66 \cdot C_3^{1/4}\lambda ^{3/4} = 0.19\;{\mathrm{nm}}$, where the spherical aberration coefficient is *C*_3_ = 0.9 mm and *λ* = 1.97 pm@300 kV is the electron wavelength. The higher spatial frequencies beyond the Scherzer focus were effectively damped by envelope functions defined by *E*_c_, which was the temporal coherency envelop caused by chromatic aberrations, focal and energy spread, etc. and *E*_a_, which was the spatial coherence envelope. Whereas the information limit and the point resolution coincide for microscopes with a thermionic electron source, the information limit goes beyond the point resolution for a FEG microscope due to the high spatial and temporal coherency. If the information limit was beyond the point resolution limit, image simulation was required to interpret details beyond point resolution. So, multiple views acquired under different focus or tilt conditions, along with quantum mechanical simulations to interpret them, were used to reconstruct the pore structure.

The HRTEM images of the pores were simulated using a multi-slice algorithm^57^. Tersely, the simulation procedure started by creating an atomistic model of the sub-nanopore topography. First, an approximation to an amorphous Si_3_N_4_ membrane was created by randomly filling a tetragonal 5 × 5 × 10 nm^3^ (*x-y-z*) cell with Si and N atoms. The total number of atoms was determined by the volume (250 nm^3^), the density of stoichiometric Si_3_N_4_ (3.44 g cm^−3^) and the molecular weight of Si_3_N_4_ (140.28 g mol^−1^). Atoms that were closer together than 0.16 nm were removed from the structure. Then, to form the sub-nanopore, atoms were selectively extracted from the membrane within a volume defined by (typically three) sections, each with a cone-angle ranging from 4° to 20° and an elliptical cross-section at the waist. The calculation of dynamic electron diffraction was then performed by partitioning the input cells into forty equidistant slices along *z*. Phase-gratings of the slices were then calculated on grids with 512 × 512 pixels in *x* and *y* for 300 kV incident electrons using the elastic and absorptive form factors (0.1 for low angle; 0.01 for high angle scattering or low Z-materials) and Debye-Waller factors (*B* = 0.00467 nm^2^) to account for the thermal motion of the atoms.

The multi-slice calculations yielded a wave-function in the exit-plane of the specimen consistent with the specified model of the pore. Based on the exit-plane wave-function, simulations of the images were constructed using a phase CTF consistent with the imaging conditions, assuming instrumental parameters for the spherical aberration coefficient, *C*_3_ = 0.9 mm, and the aperture size of the objective, 150 μm, at an acceleration voltage of 300 kV. In correspondence with the actual imaging conditions, a de-focus series ranging from −120 nm to +120 nm was calculated for comparison. The TEM image calculations account for the partial temporal coherence (*E*_c_ = 3.9) caused by chromatic aberrations, focal and energy spread, etc. and the partial spatial coherence (*E*_a_ = 0.4) caused by the finite beam convergence with a 0.4 mrad semi-angle of convergence with a focus-spread of about 4 nm.

Alternatively, a few sub-nanopores were also visualized at low beam current (<30 pA) in an aberration-corrected HAADF-STEM (FEI Themis Z) either at 300 kV or at 80 kV with a monochromator that limited the energy spread in the beam to <220 mV. Whereas the STEM resolution at 300 kV was determined to be <60 pm on a GaN lattice, the resolution at 80 kV was <120 pm according to a dumbbell lattice image acquired from (110) crystalline silicon. Regardless, the high resolution facilitated the direct interpretation of the images in terms of the mass density under the probe beam without resorting to multiple views or simulations.

### Electrolyte solutions

The electrolytic solutions were constituted from twice-polished, 18.2 ΜΩ cm de-ionized (DI) water (Simplicity 185, Millipore) and high purity, commercially available salts of NaCl (99.7% J.T. Baker), MgCl_2_ (>99.9%, Fisher), LiCl (99.99%, Aldrich), CsCl (99.999%, Alfa Aesar) and KCl (99.4%, Fisher). High purity water with a resistivity of 18.2 MΩ cm was supposed to have a neutral pH, but it was difficult to measure it directly because of the very low ionic strength. (The dissolved ions had been extracted.) Repeated attempts failed to produce a consistent measurement of the pH of DI. So, the pH of the pure DI water was not measured, but instead it was inferred to be neutral from in-line measurements of the resistivity made with the Millipore water purification system. To test the assertion about ionic strength, 100 ml of DI water was purposefully adulterated with a drop of 3 M KCl solution and then the pH was measured (Model 250, Denver Instruments, Arvada, CO) with a temperature-sensitive probe (PY-P11-2S) to be in the range 5.8–5.9. This weakly acidic pH was attributed to CO_2_ dissolved into the water. When DI water contacts air, CO_2_ can dissolve into it lowering the pH (to a value as low as 5.6). On the other hand, an electrolyte like NaCl is supposed to reduce the solubility of CO_2_ and increase the dissociation of carbonic acid, with net effect of only a slight change in pH, as evident from the drop experiment.

For the sub-nanopore conductance measurements, concentrated electrolytic solutions (0.5, 1, or 2 M) were prepared first and then aliquots were diluted to the specified concentration and de-gassed in vacuum prior to the measurement. The pH was measured (in triplicate) in similarly prepared solutions. The pH measurements were calibrated against standards at pH 4, 7, and 10 (Orion #910104, 910107, 910110, Thermo Scientific). Generally, a pH near 6 was measured in all the surrogate electrolytic solutions, which was in-line with the pH measured in DI water adulterated with a drop of KCl (Supplementary Table 4). On the other hand, the solutions of concentrated 100 mM HCl all showed pH 1.

### Estimates of electrolytic activity

In an ideal electrolytic solution of concentration *C*, ionic strength *I*, and activity *a*, the entire concentration is available for reaction, i.e. *C* = *I* = *a*, but for a non-ideal solution (especially at high >100 mM concentration), ion interactions cannot be neglected. To consider what fraction of the ions are unavailable due to ion-ion electrostatic shielding, the activity coefficient, *γ*, is determined, where the true activity is measured by *a* = γ*C* and *γ* < 1. Intuitively, *γ* depends on concentration, but it also depends on the size of the hydrated ions, *a*_0_ their charge, *z*_i_ and the relative weighting of these parameters, which is given by empirically determined values *b, A*, and *B*. Using these values, γ can be calculated by semi-empirical extended Debye-Hückel or Truesdell-Jones formuli^58^: i.e., $\log _{10}[\gamma ] = - Az_i^2(\sqrt I /[1 + Ba_i^0\sqrt I ]) + b_iI,$ depending on whether the *b*_*i*_*I*-term is included or not. Empirically, the formula extended Debye-Hückel is supposed to work best at low *I* whereas Truesdell-Jones is supposed to fit the data better at high *I*, for example. For all valence group (I) electrolytes the ionic strength *I* = *C*, but for group (II) *I* = 3 *C* due to the additional charge and also because of the additional Cl^−^ atom per molecule. Our calculations assumed values at 25 °C: i.e., *A* = 0.5085 M^−1/2^, *B* = 0.3281 × 10^−8^ M^−1/2^ m^−1^ and *a*_0_ and *b* were given in Table 1.

**Table 1:** Parameters used for the calculation of electrolyte activity

Ion:	Li	Na	K	Cs	Mg
*a*_0_ (nm)	0.38	0.36	0.33	0.33	0.43
*b* (M^−1^)	0.2	0.06	0.01	0.01	0.21

### Protein

The recombinant, carrier-free biotinylated *K*_100_ (BT-PLK100, Alamanda Polymers) homopolymer used to produce the data in Supplementary Fig. 14 was purchased as >90% pure lyophilized powder, and then re-constituted in 50 ml de-ionized water to form stock solutions of 2 mg/ml, following the protocols offered by the manufacturer. Typically, the protein was reconstituted at high (10 µg/ml) concentration in phosphate buffer saline (PBS, pH 7.4). Aliquots of these stock solutions were diluted 5000-fold with 1× PBS to produce 0.4 µg/ml for tethering. For long-term storage, the solutions were kept in 1.5 ml centrifuge tubes at −80 °C to prevent degradation, whereas for short-term (day-to-day) use, they were stored at 4 °C.

From this solution, aliquots diluted to 10× the concentration of denaturant with 300 pM protein in 250 mM of NaCl electrolyte, 1 mM β-mercaptoethanol (BME), and 0.005% (w/v) SDS were vortexed and heated to 85 °C for 1–2 h to denature the protein. To functionalize an AFM tip, the cantilever was first conditioned in a 20% oxygen plasma at 25 W (Harrick Plasma) for 1 min and then coated in a sealed container with 3-aminopropyltriethoxysilane (APTES, Gelest) by vapor deposition overnight. After this treatment, the cantilevers were stored at −20 °C for up to 10 da. Prior to a measurement, the cantilever was exposed to biotin labeled BSA (1 μg/ml, A8549, Sigma-Aldrich) in PBS for 45 min at 23 °C, rinsed with PBS and then placed in 100 μl of streptavidin (1 μg/ml, S4762, Sigma-Aldrich) in PBS for 45 min at 22 °C, rinsed in PBS and finally immersed in denatured 30 nM protein (0.5 μg/ml) in PBS and incubated for another 45 min at 23 °C followed by a final rinse in 250 mM NaCl electrolyte before mounting on the cantilever holder.

### Microfluidics

The silicon chip supporting a single membrane with a single pore through it was bonded to a polydimethylsiloxane (PDMS, Sylgard 184, Dow Corning) microfluidic device, formed using a mold-casting technique^15,16^. The microfluidic device consisted of two microchannels separated by the membrane with a pore through it: the channel on the *trans*-side was 250 × 75 μm^2^ in cross-section, whereas on the *cis*-side an 8 mm diameter reservoir was connected by a via 500 μm in diameter to the silicon chip. A tight seal was formed between the silicon chip and the PDMS *trans*-microfluidic channel with a plasma-bonding process. The membrane with a pore through it was plasma-bonded to the cis-side of the PDMS microfluidic using a (blue-white) 25 W oxygen plasma (PDS-001, Harrick Plasma, Ithaca, NY) for 30 s. The *cis*-channel was likewise sealed to a clean 75 × 25 mm^2^ glass slide, 1 mm thick (VWR, Radnor, PA) using the same bonding strategy. To ensure a tight seal to the PDMS, 3 mm diameter × 1.5 mm thick NdFeB magnets (K&J Magnetics, Pipersville, PA) were used to apply ~20 N between the silicon chip and the glass slide in a vacuum oven at 75 °C for 30 min. Subsequently, the silicon nitride layer on top of the silicon chip was painted with PDMS, and then the ensemble was again baked at a temperature of 75 °C for 30–60 min. Two separate Ag/AgCl electrodes (Warner Instruments, Hamden, CT) were embedded in each channel to independently, electrically address the *cis*-sides and *trans*-sides of the membrane. Likewise, the two microfluidic channels were also connected to external pressure and fluid reservoirs through polyethylene tubing at the input and output ports.

To test the integrity over time of the seals and electrical connections made this way, the current through a membrane without a pore through it was measured repeatedly in 250 mM NaCl and then, after flushing with 18.2 MΩ cm de-ionized (DI) water, measured again. Regardless of the electrolyte used for the measurement, a leakage current <15 pA at 0.6 V was observed for pristine membranes. Likewise, the membrane and seal integrity were measured after exposure to concentrated 100 mM HCl at pH 1. A membrane without a pore through it was measured repeatedly in 250 mM NaCl and then, after exposure to concentrated 100 mM HCl, it was flushed with DI water and measured again in 250 mM NaCl with pH 6. After repeated exposure to the acid over 4 da, the leakage current increased from <15 pA to ~45 pA at 0.6 V.

### Low-noise electrical measurements of the current and noise

To perform current measurements, first, the two microfluidic channels on the *cis*-side and *trans*-side of the membrane with a pore through it were connected to external fluid reservoirs through polyethylene tubing at the input and output ports. To remove trapped air in the microfluidic, methanol was initially flowed through the microfluidic and then immediately the channels were flushed and filled by 250 mM NaCl electrolyte. Subsequently, to wet the pore, an alternating voltage was applied for >1 da (typically). Consistent with earlier reports^14^, during electro-wetting, the pore conductance generally increased dramatically with time during the first 10 h while the rms-fluctuations in the current diminished and eventually stabilized. Whereas the leakage current was typically <15 pA (25 pS) for a pristine membrane without a pore, the sub-nanopore conductances were generally <2 nS and independent of time (>1 month).

After wetting the pore, a transmembrane voltage ranging from −0.60 V to +0.60 V was applied to the reservoir using Ag/AgCl electrodes and the corresponding open pore current was measured at 22 ± 0.1 °C using an Axopatch 200B amplifier with the output digitized by a DigiData 1440 data acquisition system (DAQ, Molecular Devices, Sunnyvale, CA) at a sampling rate of 250 kHz. Clampex 10.2 (Molecular Devices, Sunnyvale, CA) software was used for data acquisition and analysis. In a typical measurement, which took <20 min, a constant voltage bias was applied between the electrodes until a steady-state current was established, then current traces were acquired for intervals ranging from 60 s (typical) to 300 s (which was routinely used to determine the influence of the acquisition time on the accuracy of the pink noise intercept). The conductance remained constant, independent of time, at a value that corresponded to the electrical conductance of the wetting liquid for days at low molarity (<500 mM). However, at high molarity, the conductance increased dramatically within a day or two likely because the seal between the silicon chip and PDMS was compromised. To guarantee reproducibility, after each measurement, control experiments were performed using calibrated 250 mM NaCl solutions. Data was discarded if the *NaCl* conductance failed to reproduce within about 10%. Following this criterion, measurements of the conductance associated with CsCl electrolyte were frequently discarded.

### Noise estimation

To estimate the noise, the pore current traces were processed in four steps:

First, a log-log plot of the PSD as a function of frequency was used to determine the 1/*f* noise intercept *S*_1/*f*_(1 Hz) at log_10_(1 Hz). Second, the fitting of this value, *S*_1/*f*_(1 Hz) normalized by the square of the open pore current *I*_0_^2^, i.e., $S_{1/f}(1\,{\mathrm{Hz}})/I_0^2$ against *I*_0_^2^ was performed to determine the current threshold in *I*_T_ above which correlative ion motion was presumed to dominate. Third, a further linear fitting of a number of traces *n* ≥ 3 of these current thresholds *I*_T_(*a*_n_) against activity permitted extrapolation to the threshold *I*_T0_ associated with vanishingly small activity (infinite dilution). The *y*-intercept of this plot at zero-activity is the quantity of interest because it represents that current value for which the pore would exhibit correlated ion noise even at an effective concentration of zero. In this way, the current threshold was estimated at infinite dilution for a given pore and electrolyte, where supposedly no ion–ion interactions occur. Finally, this threshold was plotted as a function of pore diameter to determine the diameters (*x*-intercepts) for all ions. The *x*-intercept of this plot at *d*_*i*_ = 0 was of interest because it represents that pore diameter at which correlated ion motion would be universally observed for the ion at any *I*_0_ > 0. Thus, the limiting pore diameter for which an ion produces correlated ionic noise was associated with its confinement and was therefore a measure of its physical size as defined in detail below.

For each current trace, the data acquired within 15 s after a change in voltage was expunged to guarantee a steady-state reading of current with no capacitive influence. Two properties were then extracted from these traces; the mean open pore current *I*_0_ and the amplitude, *S*_1/*f*_(1 Hz). To determine $\log _{10}S_{1/f}(1\,{\mathrm{Hz}})$, the PSD was plotted as a function of $\log _{10}f$ and a weighted fit of the 1/*f* noise component of the trace was performed using a force fit slope of *β* = −1. The fit was preferentially weighted to low frequencies such that a hard cutoff on the higher frequency bound was unnecessary. Specifically, every two decades, the weight dropped an order of magnitude, so a PSD value at 100 Hz was 10 times less significant to the fit than the PSD recorded at 1 Hz and so on. Separately, the mean logarithmic PSD was determined in the intermediate range 1–5 KHz, where pink noise was not evident for the range of bias voltages used here. The parameter *S*_0_ was defined as the mean PSD in this range. The intercept of these two lines was found and iterated to minimize the residuals to the piece-wise fit using custom MATLAB code and produce optimal *S*_1/*f*_(1 Hz) values.

For each current trace, the quotient $S_{1/f}(1\,{\mathrm{Hz}})/I_0^2$ was calculated and plotted against *I*_0_^2^ for each electrolytic ion, concentration and sub-nanopore. As was the case for the sub-nanopore PSDs, this function exhibited two components: a power-law-dependence of power ζ = −1 for low currents (*I*_0_ ≤ 10 pA), and a component that was relatively independent of current above a threshold. Both lines were fit and the intercept of the power-law, $\sigma _ < = S_{1/f}(1\;{\mathrm{pA}})/I_0^2$ was determined. As with the PSDs, the intersection of these two lines was found and iterated to minimize the residuals to the piece-wise fit. The optimal intersection co-ordinates $[I_0^2,S_{1/f}/I_0^2]$ yielded the current threshold $(I_T^2)$ squared and *σ*_>_.

A plot of *I*_T_ against activity, *a*, for a given pore was linearly extrapolated to zero-activity to infer *I*_T0_(*a* = 0), the threshold at infinite dilution for the pore. These values were then plotted as a function of pore diameter to determine the theoretical diameter at which the threshold would be observed for any *I*_0_ > 0. As the functional form of the threshold with pore diameter was unknown, a weighted linear fit was used to determine the intercept of *I*_T0_ with pore diameter *d*_i_(*I*_T0_) over a short range. This limiting pore diameter relates the size at which correlated ionic noise dominates due to cooperative ionic motion at zero-effective-activity. These plots of current threshold against activity were generated for all ions separately, as were the resulting plots of *I*_T0_ as a function of pore size.

### Noise measurements in acurrent blockades due to protein

To perform blockade current measurements like those shown in the Supplementary Fig. 14, while systematically controlling the translocation kinetics, a denatured homopolymer, poly-l-lysine (*K*_100_), was tethered to an AFM tip and impelled through a sub-nanopore using a customized AFM (MFP-3D-BIO, Asylum Research, Santa Barbara, CA) interfaced to an inverted optical microscope (Axio-Observer Z1, Zeiss), all enclosed within a Faraday cage^16^.

To acquire the data, first the topography of the silicon nitride membrane and the location of the pore relative to the edges of the membrane were determined with a sharp tip in liquid in constant force (contact) mode. After that, the pore location was re-acquired in liquid with a second cantilever on the same probe through triangulation from the fiducial marks and a small area scan. Then a 0.7 V bias was applied across the membrane and the pore current was measured continuously at 18.0 ± 0.1 °C using an Axopatch 200B amplifier with the output digitized with the DigiData 1440 data acquisition system (DAQ, Molecular Devices, Sunnyvale, CA) at a sampling rate of 100–250 kHz, while the force on the cantilever was determined from the deflection. Starting from a position about 100–120 nm above the membrane, the tethered protein, immersed in a solution of 250 mM NaCl electrolyte and 2 × 10^−4^% (w/v) SDS, was repeatedly advanced towards the sub-nanopore at 20 nm/s, captured and threaded through it by the electric field, and then retracted from it at a constant 4 nm/s velocity by the AFM while the current, tip deflection and Z-position were recorded. The tip position above the membrane was determined from the sum of the tip deflection and Z-sensor position. Each data channel was subsequently digitally filtered at 5 kHz and sampled at 10 kHz and then digitally filtered again using a 100 Hz eight pole Bessel filter (MATLAB).

### Finite element simulations (FESs)

The FESs were performed by using COMSOL (v5.7, COMSOL Inc., Palo Alto, CA, USA). Following Luan and Stolovitzky^17^, the FESs were based on continuum modeling, which accounted for a bi-conical shape of the particular pore, the reduced electrophoretic mobility and the steric effect of ions explicitly. The electrohydrodynamics was governed by coupled Poisson and Stokes equations. Briefly, the applied potential φ and the potential *ψ* due to charges in the pore were de-coupled from one another and solved independently. The relationship between *ϕ* and the charge carriers, e.g., Na^*+*^ and Cl^*−*^, is given by the Poisson equation, ∇^2^*ψ* = −*ρ*/*εε*_0_, where *ρ*, *ε*, and *ε*_0_ were the volume charge density and the relative and vacuum permittivities, respectively. The charge density is given by $\rho = F\mathop {\sum}\nolimits_i {z_ic_i}$, where *F* = 96,485 C mol^–1^ is the Faraday constant, *z*_*i*_ is the valence and *c*_*i*_ is the molar concentrations of *i*th ionic species in the bulk. Electro-osmotic flow was captured by the Navier–Stokes equation: i.e., $\eta \nabla ^2u - \nabla p - F\mathop {\sum}\nolimits_i {z_ic_i\nabla V = 0} ,$ where the total potential, *V* = φ + *ψ*, *η* is the viscosity, *p* is the pressure and *u* is the velocity vector. The transport of ionic species is described by the Nernst–Planck equation given by: *D*_*i*_∇^2^*c*_*i*_ + *z*_*i*_*μ*_*i*_*c*_*i*_∇^2^*V* = *u* ⋅ ∇*c*_*i*_, where *D*_*i*_ is the diffusion coefficient and *μ*_i_ is the ionic mobility of the *i*th species. Thus, *u*, *V*, and *c*_*i*_ are coupled between equations. The boundary conditions are specified in the Supplementary Table 1 and material properties such as the diffusivity in the sub-nanopore were restricted by the literature^18,19,35–37^.

To estimate the pore conductance, the radial distributions of the electric potentials and ion concentrations were calculated, assuming that the radius-dependent concentration followed a Boltzmann distribution according to: $\nabla ^2\Psi = \sinh (\Psi )/\lambda ^2(1 + \alpha (\cosh (\Psi ) - 1))$, Ψ = *eψ*/*k*_*B*_*T*, where where *ψ* is the (radius-dependent electric potential), *k*_B_ is the Boltzmann constant; *T* is the absolute temperature; and *l* is the Debye screening length and *α* = 2*a*^3^*n*_0_, where *a* is the radius of the solvated ion.

The topography of each pore was taken into account to match the data. With the assumption of a pore topography, the effective thickness of the membrane was estimated from the electric field distribution using FESs. With this estimate, the surface charge density was then inferred from measurements of the pore conductance at dilute concentration, assuming initially that the cations carried the current prodominately and that the electro-osmotic flow was negligible. With all of these assumptions, the cation diffusity (constrained by the literature) was then extracted by matching the empirical results acquired with a concentration gradient (250 mM/1 mM) imposed across the membrane first at *V* = 0 V and then at *V* = 0.6 V. Finally, to assess the anionic contribution to the current, the data acquired in an electrolyte gradient were fit at *V* = −0.6 V. With these parameters the entire range of the concentration dependence of the conductace was fit and then the algorithm was iterated until the parameters converged. Finally, with these parameters in hand, the current–voltage characteristics measured without a gradient imposed across the membrane were then matched.

The temperature rise resulting from Joule heating was inferred from FESs governed by the heat equation: $\rho C_p\frac{\partial }{{\partial t}}T({\bf{r}},t) = \nabla \cdot [\kappa \nabla T] + {\bf{J}}({\bf{r}},t) \cdot {\bf{E}}({\bf{r}},t)$, which included a source term: **J**(**r**,*t*) ⋅ **E**(**r**, *t*) = *σ*(*T*, **r**, *t*) ⋅ **E**(**r**, *t*) ⋅ **E**(**r**, *t*), where *T* represents the temperature, *ρ*, *σ, C*_*p*_ and *κ* denote the density, temperature-dependent electrical conductivity, heat capacity and thermal conductivity, respectively (Supplementary Tables 1, 2) and **E**(**r**, *t*) = −∇*V*(**r**, *t*), where *V*(**r**, *t*) denotes the applied voltage following other work^38^. In conjunction with the heat equation, current continuity ∇ ⋅ **J**(**r**, *t*) = ∂*ρ*_*c*_(**r**, *t*)/∂*t* and the Poisson’s ∇ ⋅ [*ε*(**r**, *t*) ⋅ **E**(**r**, *t*)] = *ρ*_*c*_(**r**, *t*) and the constitutive relation **J**(**r**, *t*) = *σ*(*T*, **r**, *t*) ⋅ **E**(**r**, *t*) were used to specify the solution. Although the electrolyte and membrane properties were temperature-dependent, they were considered as constants for the range of voltage and current tested here (Supplementary Table 2). The IAPWS-95 formulation for the equation of state of the water was used to determine the temperature-dependent characteristics of 250 mM NaCl electrolyte.

### Molecular dynamics (MD) simulations

For an accurate assessment with atomic detail, following earlier work^14^, the ion transport was simulated in sub-nanopores through a Si_3_N_4_ membrane by MD. All the simulations were performed using GROMACS 4.6.7 in an NVT ensemble^59^. To construct the sub-nanopore, a cubic unit cell of α-Si_3_N_4_ crystal was first replicated in three dimensions to produce a cubic box that formed the membrane. For economy, an idealized cylindrical sub-nanometer-diameter channel 2.5 nm long was produced by removing atoms from α-Si_3_N_4_ membrane according to the criterion $\sqrt {(x - x_c)^2 + (y - y_c)^2} < r + \delta$ (regardless of their *z*-coordinates), where (*x*, *y*) is the coordinate of the atom, (*x*_c_, *y*_c_) is the coordinate of the center of the pore, and *δ* is the van der Waals radii of the pore surface atoms (taken as 0.16 nm). The conical shape near each orifice in the actual pore topography was faithfully mimicked extending from each side of the channel (Fig. 1f). In the simulations, the van der Waals interactions between atoms were modeled as (6,12) Lennard–Jones atoms^60^ with CHARMM force fields for the Si and N atoms in membrane^61^. Water was modeled with the SPC/E model^62^.

A Si_3_N_4_ surface immersed in an electrolyte solution usually carries a net surface charge. In this work, we assume a negative surface charge density of *σ*_s_ = −0.125 e nm^*−*2^ on the pore surface, which is typical of a Si_3_N_4_ surface immersed in a solution with neutral pH. To represent the net surface charge density, charges were then added to the surface atoms of the pore. When a charge of *Q* was assigned to a pore, each of the surface atoms had an equal charge of *Q*/(number of surface atoms). The additional charge was typically balanced by an excess of counter-ions in the simulation system.

The electrostatic interactions were computed by using the Particle–Mesh–Ewald method with no truncation for the Coulomb interactions. A cutoff distance of 1.10 nm was used in the calculation of electrostatic interactions in the real space. A fast Fourier transform grid spacing of 0.11 nm and cubic interpolation for charge distribution were chosen to compute the electrostatic interactions in the reciprocal space. The system temperature was regulated at 300 K by using a Nośe–Hoover thermostat. The equation of motion was integrated by using the leap-frog algorithm with a time step of 1.0 fs. Usually, starting from a random configuration, the system was simulated for 1 ns to reach a steady state, followed by a production run exceeding >50 ns. The ion distribution in the pore was computed by using the binning method, and the ion velocity was computed by tracking the positions of the ions.  The PMF for an ion *j*, *W*_*j*_(*z*), was computed by integrating the mean force acting on ion j along the nanopore axis, *z*:

1 $$W_j(z) - W_j(z_0) = \mathop {\int}\limits_{z_0}^z {\left\langle {F_j(z\prime )} \right\rangle {\mathrm{d}}z\prime }$$

where the mean force $\left\langle {F_j(z{\prime})} \right\rangle ,$, was obtained by accounting for all the atoms in the system averaged over all the configurations and *z*_0_ is the reference position (*W*_*j*_(*z*_0_) = 0), and was taken as the position where the mean force was zero.

The ion diffusivity was computed as a slope of the mean square displacement (MSD):

2 $$D = \mathop {{\lim }}\limits_{t \to \infty } \frac{{\left\langle {[R_j(t) - R_j(0)]^2} \right\rangle }}{{6t}}$$

where *R*_*j*_(*t*) is the position of ion *j*. Equilibrium MD was also performed for ions in a pore without any electric field, since the diffusivity is defined at equilibrium. Then, the ion mobility was calculated via the Einstein relation. Both the average and instantaneous ionic currents were extracted from MD simulation data. The average ionic current was defined as:

3 $$I = \frac{{\left( {{\mathrm{Total}}\,{\mathrm{number}}\,{\mathrm{of}}\,{\mathrm{counter}} - {\mathrm{ions}}\,{\mathrm{translocating}}} \right) \times e}}{{{\mathrm{Total}}\,{\mathrm{simulation}}\,{\mathrm{time}}}},$$

where as the instantaneous ionic current was expressed:

4 $$I(t) = \mathop {\sum}\limits_j^{{\mathrm{Number}}\,{\mathrm{of}}\,{\mathrm{translocations}}} {\left( {\frac{{{\mathrm{Unit}}\,{\mathrm{charge}}\,(e)}}{{{\mathrm{Translocation}}\,{\mathrm{time}}\,{\mathrm{through}}\,{\mathrm{pore}}}}} \right)} ,$$

which focused on each event of an ion translocation. These formulae estimate how many ions traveled through a sub-nanopore in an interval of time, Δ*t*. The simulations focused exclusively on the monovalent ions (Li^+^, Na^+^, and Cl^−^), and therefore the unit charge, *e*, was multiplied by the total number of ion translocations to compute the charge transfer through the pore during Δ*t*. Since the pore diameter was as small as *d* = 0.30 and 0.50 nm, the co-ions did not enter the pore and so the co-ions were precluded from contributing to the ionic current. Once the average current was calculated, the corresponding ionic conductance was estimated as the slope in the current–voltage characteristic.

## Supplementary information

Supplementary Information

## Data Availability

Summaries of the data generated and/or analyzed during the current study are included in the published article and the corresponding supplementary information file. These data are available from the corresponding author on reasonable request.

## References

[1] Beckstein O, Sansom MSP (2004). The influence of geometry, surface character, and flexibility on the permeation of ions and water through biological pores. Phys. Biol..

[2] Esfandiar A (2017). Size effect in ion transport through angstrom-scale slits. Science.

[3] Chmiola J (2006). Anomalous increase in carbon capacitance at pore sizes less than 1 nanometer. Science.

[4] Bakker HJ, Kropman MF, Omta AW (2005). Effect of ions on the structure and dynamics of liquid water. J. Phys. Condens. Matter.

[5] Ansell S, Barnes AC, Mason PE, Neilson GW, Ramos S (2006). X-ray and neutron scattering studies of the hydration structure of alkali ions in concentrated aqueous solutions. Biophys. Chem..

[6] Burikov SA, Dolenko TA, Fadeev VV, Vlasov II (2007). Revelation of ion hydration in Raman scattering spectral bands of water. Laser Phys..

[7] Mähler J, Persson I (2012). A study of the hydration of alkali metal ions in aqueous solution. Inorg. Chem..

[8] Li H, Francisco JS, Zeng XC (2015). Unraveling the mechanism of selective ion transport in hydrophobic subnanometer channels. Proc. Natl Acad. Sci. USA.

[9] Smith DW (1977). Ionic hydration enthalpies. J. Chem. Educ..

[10] Choi W (2013). Diameter-dependent ion transport through the interior of isolated single-walled carbon nanotubes. Nat. Commun..

[11] Raviv U, Laurat P, Klein J (2001). Fluidity of water confined to subnanometre films. Nature.

[12] Levinger NE (2002). Water in confinement. Science.

[13] Fumagalli L (2018). Anomalously low dielectric constant of confined water. Science.

[14] Ho C (2005). Electrolytic transport through a synthetic nanometer-diameter pore. Proc. Natl Acad. Sci. USA.

[15] Kennedy, E., Dong, Z., Tennant, C. & Timp, G. Reading the primary structure of a protein with 0.07 nm^3^ resolution using a sub-nanometre-diameter pore. *Nat. Nanotechnol*. **11**, 968–976 (2016). 10.1038/nnano.2016.120 27454878

[16] Dong Z, Kennedy E, Hokmabadi M, Timp G (2017). Discriminating residue substitutions in a single protein molecule using a sub-nanopore. ACS Nano.

[17] Luan B, Stolovitzky G (2013). An electro-hydrodynamics-based model for the ionic conductivity of solid-state nanopores during DNA translocation. Nanotechnology.

[18] Ortiz-Young D, Chiu HC, Kim S, Voïtchovsky K, Riedo E (2013). The interplay between apparent viscosity and wettability in nanoconfined water. Nat. Commun..

[19] Gao J, Szoszkiewicz R, Landman U, Riedo E (2007). Structured and viscous water in sub-nanometer gaps. Phys. Rev. B.

[20] Lizana L, Ambjörnsson T (2009). Diffusion of finite-sized hard-core interacting particles in a one-dimensional box: tagged particle dynamics. Phys. Rev. E.

[21] Feng J (2016). Observation of ionic Coulomb blockade in nanopores. Nat. Mater..

[22] Detcheverry F, Bocquet L (2012). Thermal fluctuations in nanofluidic transport. Phys. Rev. Lett..

[23] Zorkot M, Golestanian R, Bonthuis DJ (2016). The power spectrum of ionic nanopore currents: the role of ion correlations. Nano Lett..

[24] Weissman MB (1988). 1/*f*-noise and other slow, non-exponential kinetics in condensed matter. Rev. Mod. Phys..

[25] Zandbergen HW, Van Dyck D (2000). Exit wave reconstructions using through-focus series of HREM images. Microsc. Res. Tech..

[26] Volkov AG, Paula S, Deamer DW (1997). Two mechanisms of permeation of small neutral molecules and hydrated ions across phospholipid bilayers. Bioelectrochem. Bioenerg..

[27] Kiriukhin MY, Collins KD (2002). Dynamic hydration numbers for biologically important ions. Biophys. Chem..

[28] Shannon RD (1976). Revised effective ionic radii and systematic studies of interactomic distances in halides and chalcogenides. Acta Crystallogr..

[29] Marcus Y (1988). Ionic radii in aqueous solutions. Chem. Rev..

[30] Carrillo-Tripp M, Saint-Martin H, Ortega-Blake I (2004). Minimalist molecular model for nanopore selectivity. Phys. Rev. Lett..

[31] Apel PY, Blonskaya IV, Orelovtch OL, Ramiriz P, Sartowska BA (2011). Effect of nanopore geometry on ion current rectification. Nanotechnology.

[32] Mouterde T (2019). Molecular streaming and its voltage control in angstrom-scale channels. Nature.

[33] Queralt-Martin M, López ML, Aguilella-Arzo M, Aguilella VM, Alcaraz A (2018). Scaling behavior of ionic transport in membrane nanochannels. Nano. Lett..

[34] Borukhov I, Andelman D, Orland H (1997). Steric effects in electrolytes: a modified Poisson-Boltzmann equation. Phys. Rev. Lett..

[35] Lynden‐Bell RM, Rasaiah JC (1996). Mobility and solvation of ions in channels. J. Chem. Phys..

[36] Zhou JD, Cui ST, Cochran HD (2003). Molecular simulation of aqueous electrolytes in model silica nanochannels. Mol. Phys..

[37] Smith GR, Sansom MSP (1998). Dynamics properties of Na^+^ ions in models of ion channels: a molecular dynamics study. Biophys. J..

[38] Levin EV, Burns MM, Golovchenko JA (2016). Nanoscale dynamics of Joule heating and bubble nucleation in a solid-state nanopore. Phys. Rev. E.

[39] Maxwell JC (1904). A Treatise on Electricity and Magnetism.

[40] Sahu S, Zwolak M (2018). Maxwell-Hall access resistance in graphene nanopores. Phys. Chem. Chem. Phys..

[41] Marcus Y (2012). Volumes of aqueous hydrogen and hydroxide ions at 0 to 200 °C. J. Chem. Phys..

[42] Peng Y, Swanson JM, Kang SG, Zhou R, Voth GA (2015). Hydrated excess protons can create their own water wires. J. Phys. Chem. B.

[43] Bermudex VM (2005). Wet-chemical treatment of *Si*_*3*_*N*_*4*_ surfaces studied using infrared attenuated total reflection spectroscopy. J. Electrochem. Soc..

[44] Hoogerheide DP, Garaj S, Golovchenko JA (2009). Probing surface charge fluctuations with solid-state nanopores. Phys. Rev. Lett..

[45] Jain T (2015). Heterogeneous sub-continuum ionic transport in statistically isolated graphene nanopores. Nat. Nanotechnol..

[46] Bezrukov SM, Kasianowicz JJ (1993). Current noise reveals protonation kinetics and number of ionizable sites in an open protein ion channel. Phys. Rev. Lett..

[47] Zwolak M, Lagerqvist J, Di Ventra M (2009). Quantized ionic conductance in nanopores. Phys. Rev. Lett..

[48] Richards LA, Schafer AI, Richards BS, Corry B (2012). The importance of dehydration in determining ion transport in narrow pores. Small.

[49] Yoo J, Aksimentiev A (2015). Molecular dynamics of membrane-spanning DNA channels: conductance mechanism, electro-osmotic transport and mechanical gating. J. Phys. Chem. Lett..

[50] Dimitrov V (2010). Nanopores in solid-state membranes engineered for single molecule detection. Nanotechnology.

[51] Smeets RMM, Keyser UF, Dekker NH, Dekker C (2008). Noise in solid-state nanopores. Proc. Natl Acad. Sci. USA.

[52] Tasserit C, Koutsioubas A, Lairez D, Zalczer G, Clochard MC (2010). Pink noise of ionic conductance through single artificial nanopores revisited. Phys. Rev. Lett..

[53] Weissman MB (1975). Simple model for 1/*f*-noise. Phys. Rev. Lett..

[54] Dechant A, Lutz E (2015). Wiener-Khinchin theorem for nonstationary scale-invariant process. Phys. Rev. Lett..

[55] Liu AJ, Nagel SR (1998). Nonlinear dynamics: jamming is not just cool any more. Nature.

[56] Morgan BJ (2017). Lattice-geometry effects in garnet solid electrolytes: a lattice-gas Monte Carlo simulation study. R. Soc. Open Sci..

[57] Barthel, J. Dr. Probe-High-resolution (S)TEM image simulation software, version 1.6 http://www.er-c.org/barthel/drprobe (2015). 10.1016/j.ultramic.2018.06.003 29906518

[58] Truesdell AH, Jones BF (1974). WATEQ—A computer program for calculating chemical equilibria of natural waters. J. Res. US Geol. Surv..

[59] Hess B, Kutzner C, van der Spoel D, Lindahl E (2008). GROMACS 4: algorithms for highly efficient, load-balanced, and scalable molecular simulation. J. Chem. Theory Comput..

[60] Koneshan S, Rasaiah JC, Lynden-Bell RM, Lee SH (1998). Solvent structure, dynamics, and ion mobility in aqueous solutions at 25 °C. J. Phys. Chem. B.

[61] Huang J, MacKerell AD (2013). CHARMM36 all-atom additive protein force field: validation based on comparison to NMR data. J. Comput. Chem..

[62] Berendsen HJC, Grigera JR, Straatsma TP (1987). The missing term in effective pair potentials. J. Phys. Chem..

