# Supplementary material for: Measurements of the size and correlations between ions using an electrolytic point contact
Source: Nat Commun. 2019 May 30;10:2382. doi: 10.1038/s41467-019-10265-2 (PMC6542849; doi:10.1038/s41467-019-10265-2)
Supplement: Supplementary file 1 — Supplementary Information [file 41467_2019_10265_MOESM1_ESM.pdf]

SUPPLEMENTARY INFORMATION
Measurements of the Size and Correlations between Ions using an Electrolytic Point Contact

Eveline Rigo[#1], *Zhuxin Dong*[#1], *Jae Hyun Park*,[2] *Eamonn Kennedy*[1],
Mohammad Hokmabadi[1], *Lisa Almonte-Garcia*[1], *Li Ding*[1], *Narayana Aluru*[3], *Gregory Timp**[1]
[1]Electrical Engineering and Biological Science, University of Notre Dame, Notre Dame, IN 46556 USA
[2]Department of Aerospace and Software Engineering and Research Center for Aircraft Parts Technology, Gyeongsang National University, Jinju, Gyeongnam 52828, Republic of Korea
[3]Mechanical Engineering, University of Illinois, Urbana, IL 61801 USA

[#]These authors contributed equally.
*Correspondence should be addressed to G.T. (email: gtimp@nd.edu)

SUPPLEMENTARY FIGURE 1.

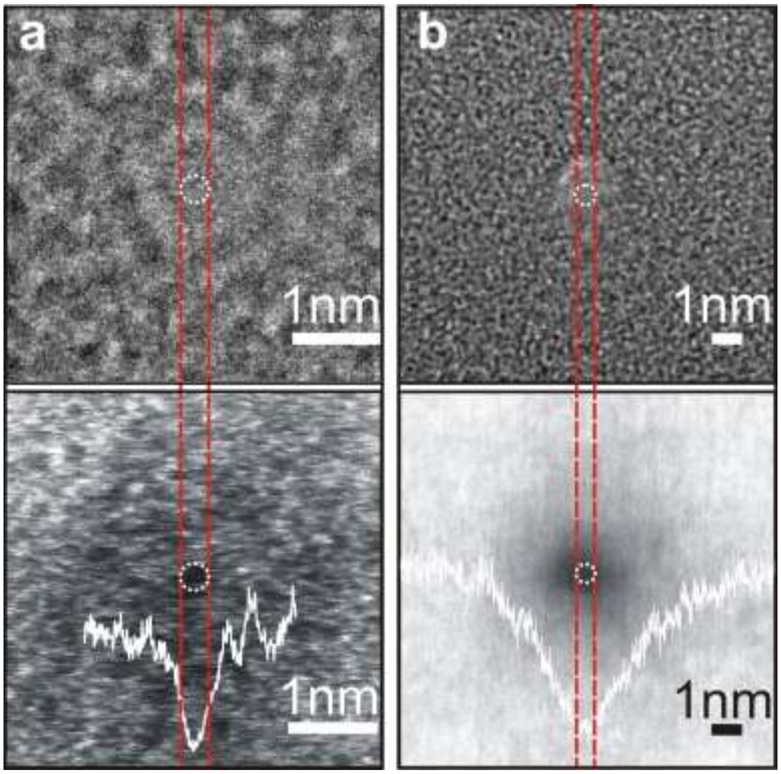

SUPPLEMENTARY FIGURE 1. The same sub-nanopores visualized with High Resolution Transmission Electron Microscopy (HRTEM) and aberration-corrected, High-angle Annular Dark Field (HAADF-) Scanning TEM (STEM). (a; top) A sub-nanopore was sputtered using the FEI Titan in STEM, and then visualized *in situ* with HRTEM with an information-limit of 0.11 nm and a point resolution < 0.2 nm. The shot noise bounded by the red-dashed box indicates the size of the pore; the diameter (white dotted circle) was estimated to be about with a 0.30 nm. (a; bottom) The same pore was subsequently re-acquired in an FEI Themis Z aberration-corrected STEM with an illumination half-angle of 18.0 mrad and an energy width of about 1.2 V and then imaged at 300 kV with HAADF at 60 pm resolution. The white-solid line trace juxtaposed on the image indicates the mass-density under the beam. (b; top) Like (a; top), using a sub-nanopore with a diameter (white dotted circle) that was estimated to be about with a 0.59 nm. (b; bottom)

Like (a; bottom), but for the sub-nanopore shown in (b;top) instead. The sub-nanopore was subsequently re-acquired in an FEI Themis Z aberration-corrected STEM with an illumination half-angle of 27.1 mrad, using a monochromator to limit the energy dispersion in the beam to 200 mV, and imaged at 80 kV using HAADF with 120 pm resolution. The white-solid line trace juxtaposed on the image indicates the mass-density under the beam.

SUPPLEMENTARY FIGURE 2.

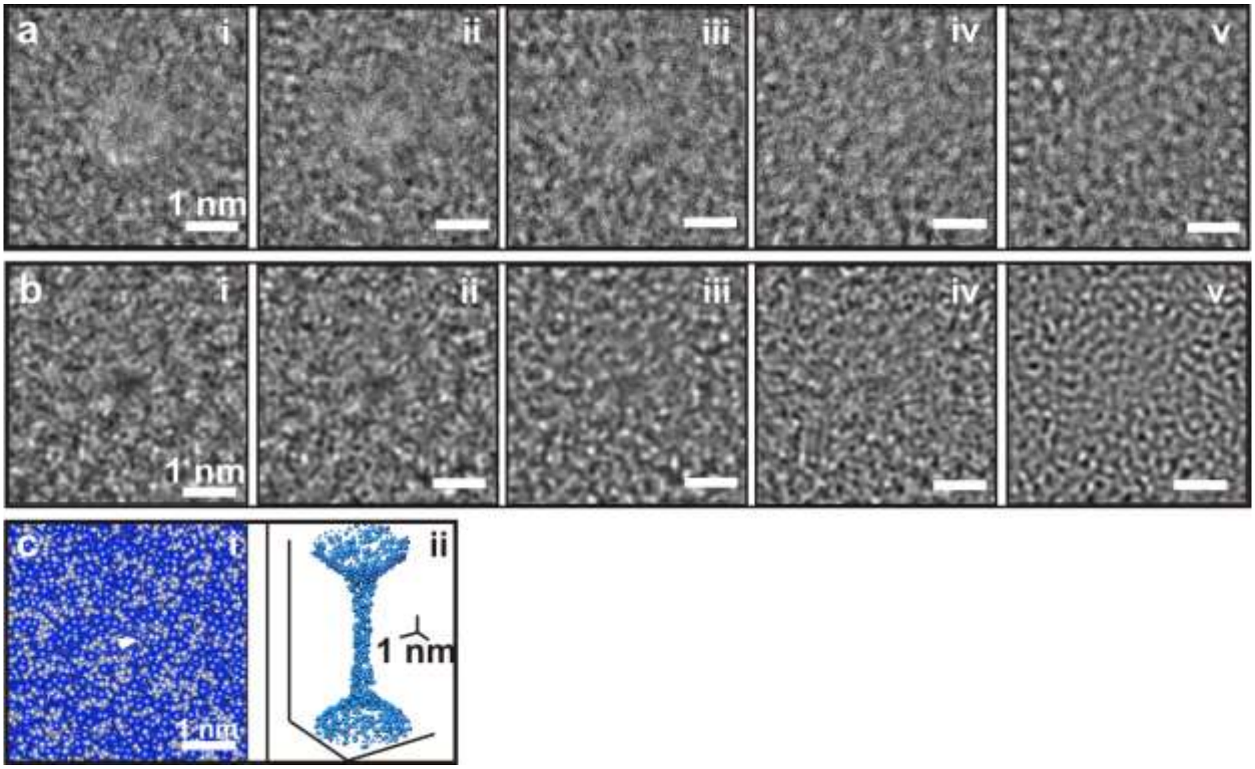

SUPPLEMENTARY FIGURE 2. A sub-nanopore visualized with High Resolution Transmission Electron Microscopy (HRTEM) and multi-slice simulations. (a; i-v) Actual TEM micrographs of the same sub-nanopore are shown for a series of defocus conditions: +96, +72, +48, +24 and 0 nm, respectively, which were used to infer the pore topography. The sub-nanopore, which had a geometric mean-diameter of 0.42 nm at the waist, was sputtered through a silicon nitride membrane that was nominally 10 nm thick. The shot noise near the center of the pore was associated with electron transmission through the pore. (b; i-v) Like (a), but showing the corresponding multi-slice simulations of the TEM images, which were consistent with the imaging conditions. These simulations assume a bi-conical pore with a 2° cone angle and a 0.35 × 0.50 nm^2 cross-section at the waist. The close correspondence between the simulations and the actual TEM images indicated that the model accurately reflected the pore topography. (c; i) A two-dimensional projection from the top through the model associated with +72 nm defocus is shown, which indicates the atomic distribution near the pore waist. The atoms are represented by a space-filling model in which *Si* is represented by a (blue) sphere with a 0.235 nm-diameter and *N* by a (gray) sphere with a 0.130 nm-diameter. (c; ii) A three-dimensional perspective of space-filled representations of the pore models. For clarity, only atoms on the pore surface are shown.

SUPPLEMENTARY FIGURE 3.

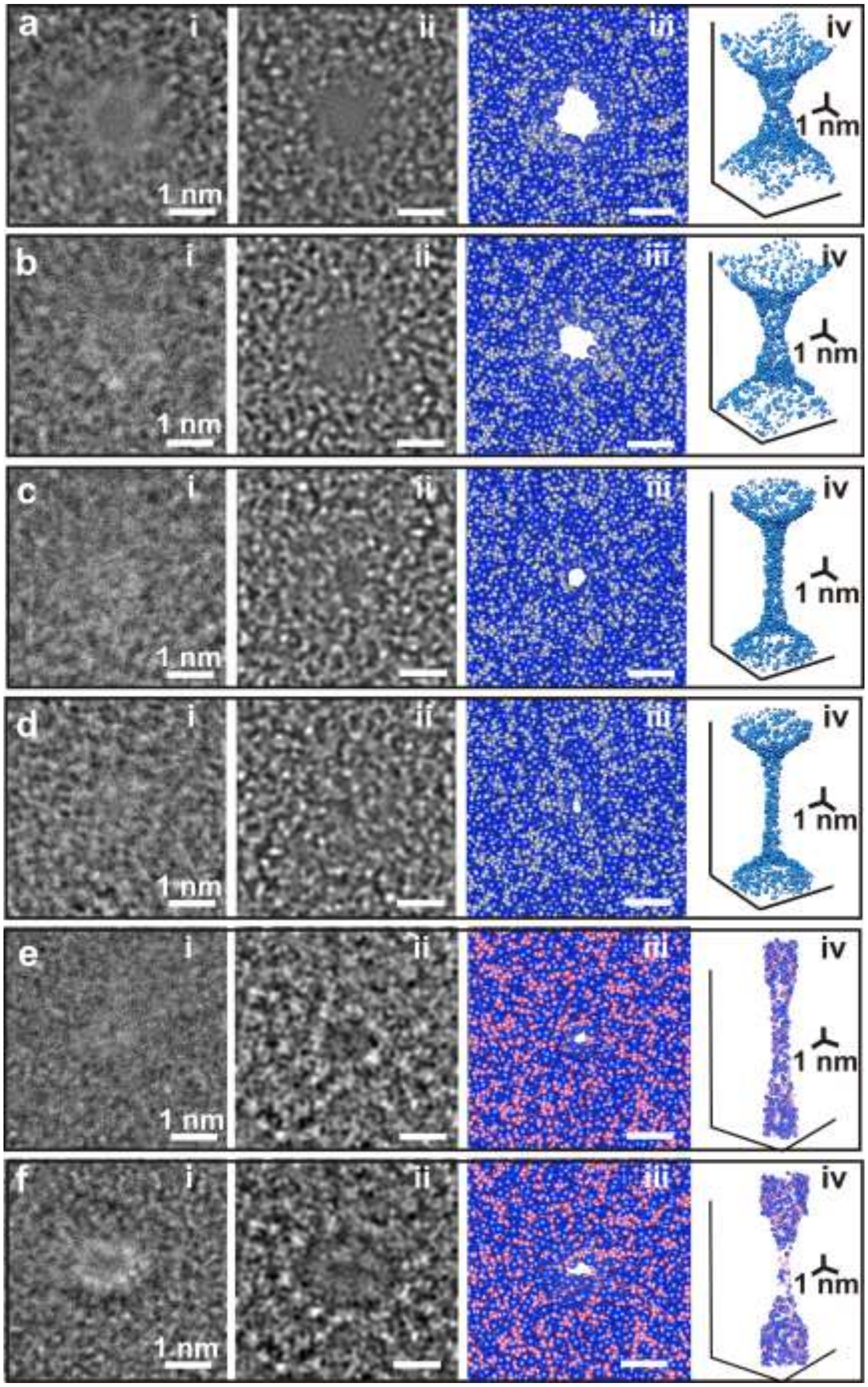

SUPPLEMENTARY FIGURE 3. The topography of sub-nanometer-diameter pores visualized with High Resolution Transmission Electron Microscopy (HRTEM) and multi-slice simulations. (a-f; i) TEM images that epitomize the sub-nanopore topographies used in this work. The pores were sputtered through nominally 10 nm thick silicon nitride membranes with (geometric) mean-diameters at the waist of 0.97 nm, 0.82 nm, 0.52 nm, 0.32 nm, 0.24 nm, and (0.28 × 0.56 nm =) 0.40 nm, respectively. (a-f; ii) The corresponding multi-slice simulations of the structures shown in (a-f; i) that were consistent with the imaging conditions (40–52 nm de-focus). The simulations generally assumed a structure with a graduated bi-conical topography with cone-angles ranging from 45° to 10° near the orifices and 10° to 2° near the waist. (a-f; iii) Two-dimensional

projections from the top through the model are shown indicating the atomic distributions near the pore waist. The atoms are depicted by space-filling models in which each *Si* is a blue sphere with a 0.235 nm-diameter and each *N* is a gray sphere with a 0.130 nm-diameter. (a-f; iv) A three-dimensional perspective of the space-filled representations of the pore models is shown. For clarity, only atoms on the pore surface are shown. The close correspondence between the simulations and the actual TEM images indicated that the models accurately reflected the actual pore topography.

SUPPLEMENTARY FIGURE 4.

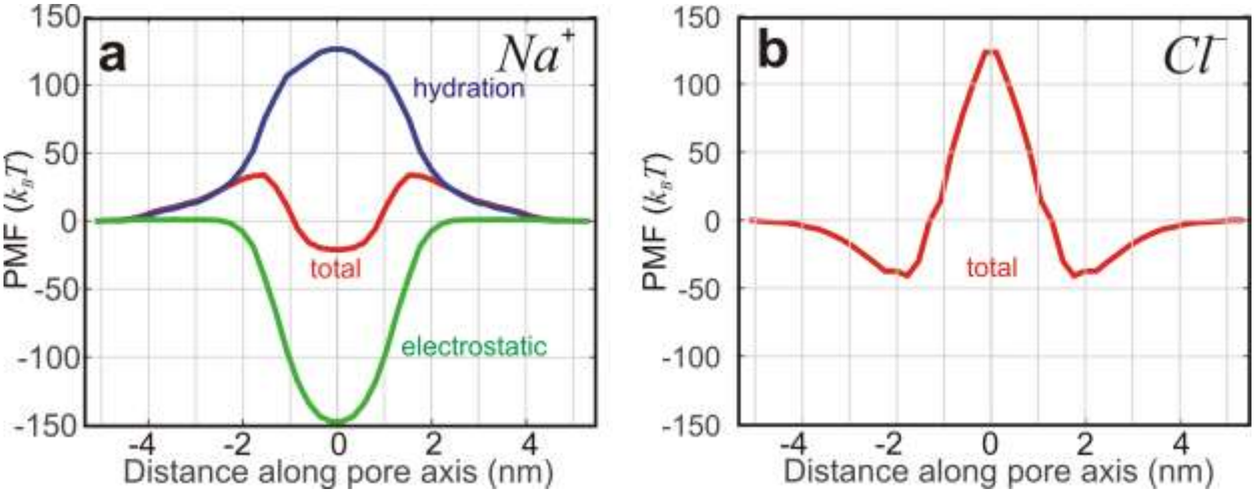

SUPPLEMENTARY FIGURE 4. Potential Mean Force (PMF) on ions in a sub-nanopore. (a,b) A plot is shown of the PMF (red lines) for Na^+/Cl^-ions respectively in a sub-nanopore with total charge –3.0*e* distributed between all the surface atoms. The total PMF (red lines) can be analyzed into ion-water interactions (denoted at hydration, blue line) and ion-pore interactions (denoted as electrostatic, green line). The plot (a) reveals a low-energy region located within the pore for the counter-ion Na^+, which implies that once it surmounts the energy barrier at the entrance, the cation can remain stably within the pore. Since the hydration shell of Na^+ is peeled off as the cation enters the pore, the *Na*-water interaction repels the ion, forming the energy barrier. However, the electrostatic attraction lowers the barrier impelling the Na^+ ion into the pore. On the other hand, the PMF for Cl^- shown in (b) is significantly larger due to strong repulsion between the anion and the negative surface charge in the pore.

SUPPLEMENTARY FIGURE 5.

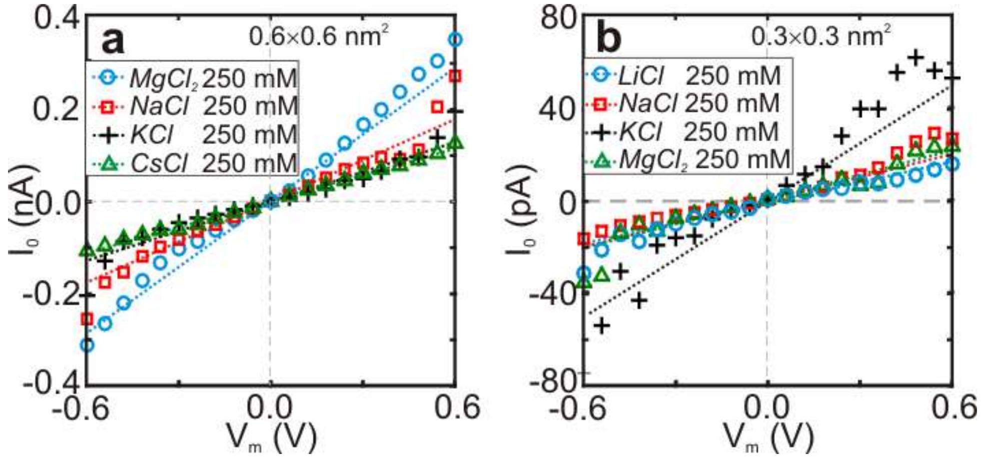

SUPPLEMENTARY FIGURE 5. Electrolytic ion transport through sub-nanopores. **(a,b)** Juxtapositions of the measured current-voltage characteristics are shown, which were acquired, respectively, from pores with 0.6 nm and 0.3 nm mean-diameters at the waist for different electrolytes at the same bulk concentration (250 mM).

SUPPLEMENTARY TABLE 1. Parameters used in Finite Element Simulations (FESs) of the sub-nanopore thermal conductance and temperature distributions.

**Boundary**	**Boundary Conditions**
**1: pore**	d = 0.3, 0.5, 0.8 and 1 nm diameter,<br>L_m = 10 nm thick membrane,<br>10° and 20° bi-conical structure
**2: *Si_3N_4* membrane**	the surface charge $\sigma_s < -0.025$ $e^-\cdot nm^{-2}$;<br>$\varepsilon_r = 7.5$
**3: Electrolyte**	250mM *NaCl*; $\varepsilon_r = 78.5$;
**4: *cis*-side boundary**	$\phi = 0.00 - 0.60V$; $\sigma = 0$; $p = 0$; $c_i = c_0$

SUPPLEMENTARY TABLE 2. Parameters used in FESs of the sub-nanopore thermal conductance and temperature distributions.‡

**Material**	$\rho(kg/m^3)$	$C_P(J/kg\cdot°K)$	$\kappa(W/m\,°K)$	$\sigma(S/m)$	ε_r
Si_3N_4	3100	700	3.2	0	7.5
Si	2329	700	130.	10	11.7

‡Data were taken from reference 1.

SUPPLEMENTARY NOTE #1: Simulations of the temperature in a sub-nanopore.

To support the assertion that the relatively small electrolytic current density in the pore waist $(J < 1\times10^5\,A/cm^2)$ precluded Joule heating, the temperature was inferred from FESs governed by the heat equation (see Methods). Although the electrolyte and membrane properties are temperature-dependent, they were deemed to be independent of temperature for the ranges of

voltages and currents tested here. After matching the current-voltage characteristic, at a 0.60 V transmembrane voltage with the sub-nanopore immersed in a 250 mM *NaCl* electrolyte, the simulation revealed a temperature distribution that peaked in the waist of a 0.30 nm-diameter pore at 293.15 K, representing practically no change from the ambient (Supplementary Fig. 6d; i, iii). Likewise, pores with a large diameter, even at 1 V, showed hardly any increase in temperature.

SUPPLEMENTARY FIGURE 6.

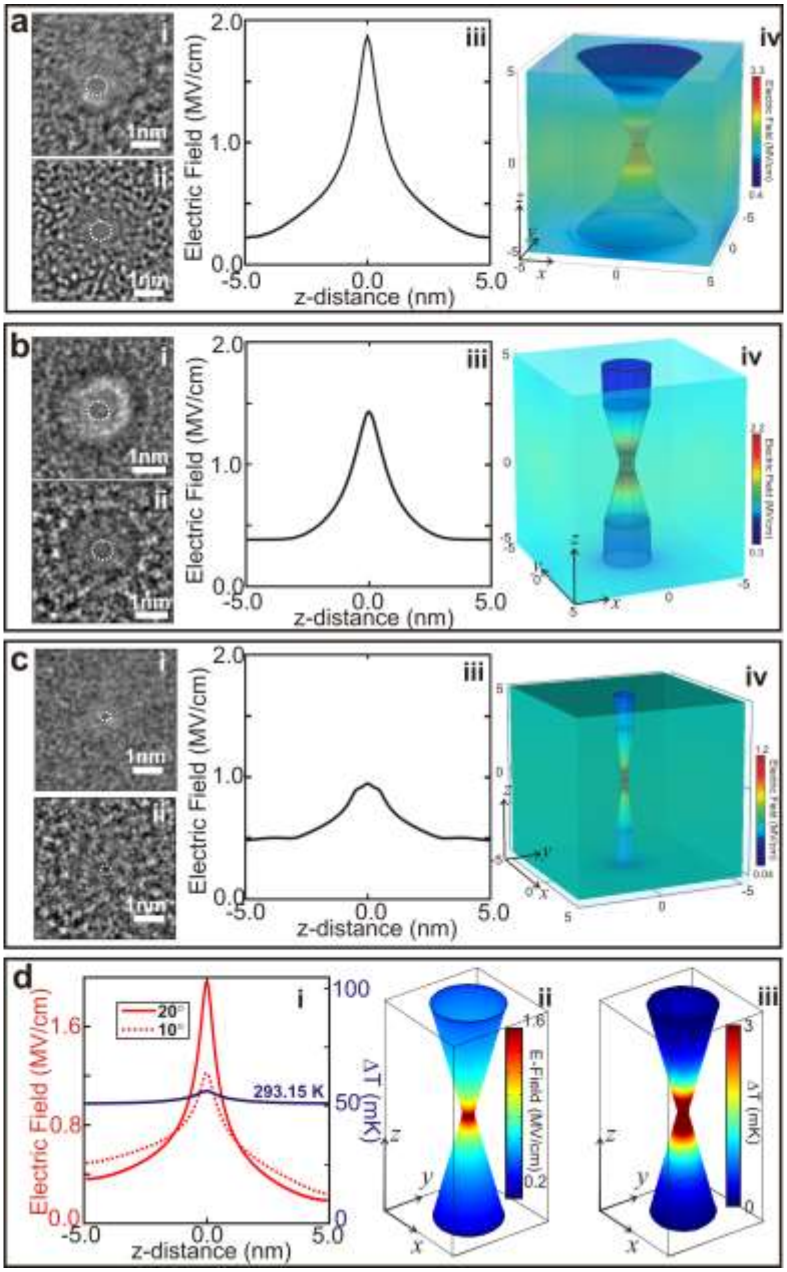

SUPPLEMENTARY FIGURE 6. Finite Element Simulations (FES) of the electric field and temperature distribution in sub-nanopores. (a-c;i) Transmission Electron Microscopy (TEM)

images that summarize the sub-nanopore topographies used in this work. The pores were sputtered through nominally 10 nm thick silicon nitride membranes with (geometric) mean-diameters at the waist of 0.70 nm, 0.80 nm, and 0.25 nm, respectively. (a-c; ii) The corresponding multi-slice simulations of the structures are shown, representing two-dimensional projections from the top through the models that match the actual images shown in (a-c; i). The simulations were consistent with the imaging conditions (40 – 52 nm de-focus). The atoms are depicted by space-filling models in which each *Si* is a blue sphere with a 0.235 nm-diameter and each *N* is a gray sphere with a 0.130 nm-diameter. (a-c; iii) FESs are shown of the electric field and temperature distribution along the vertical *z*-axis of the sub-nanopore shown in (i) with a bi-conical structure through a nominally 10 nm thick silicon nitride membrane immersed in 250 mM *NaCl* at 0.6 V bias. (a-c; iv) Superimposed on a model of the pore topography are shown heat-maps of the electric field distributions. (d;i) FESs are shown of the electric field and temperature distribution along the vertical *z*-axis of a pore with a 0.4 nm-diameter at the waist and bi-conical structure with a 10/20° cone-angle through a nominally 10 nm thick silicon nitride membrane immersed in 250 mM *NaCl* at 0.6 V bias. The field is focused over an extent of 1.5 nm near the pore waist, but regardless the temperature rise is negligible. (d; ii,iii) Superimposed on a model of the pore topography are shown heat-maps of the field and temperature distribution similar to the pore in (i), but with a 10° cone-angle.

SUPPLEMENTARY FIGURE 7.

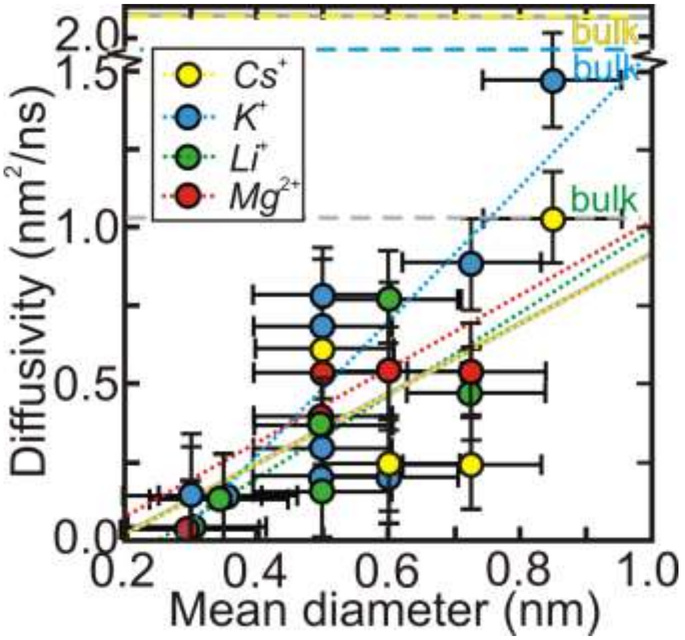

SUPPLEMENTARY FIGURE 7. Cation diffusivity extracted from Finite Element Simulations (FES) of the sub-nanopore conductance. The dependences of the diffusivity extracted from FES matches to the data on the geometric mean-diameter of sub-nanopores are shown for the four cations (Cs^+, K^+, Li^+, and Mg^{2+} acquired at concentrations ranging from 125 to 500 mM. The Cs^+, K^+, Li^+, and Mg^{2+} diffusivities extrapolate to zero near pore mean-diameters of 0.18, 0.28, 0.26 and 0.12 nm, respectively.

SUPPLEMENTARY NOTE #2: The sub-nanopore conductance depends on the specific ion.

Interestingly, the specific cation (whether hydrated or not) affected the conductance in a subtle way. This effect was first apparent in the dispersion of the conductance between different electrolytes (Figs. 2c,f and Supplementary Figs. 5-7). According to prior work,[2-7] the diameter of hydrated cations were supposed to range from 0.658 nm (Cs^+) up to 0.856 nm (Mg^{2+}), whereas the de-hydrated diameters were supposed to show a larger relative standard deviation (RSD): i.e. 0.144 nm (Mg^{2+}) to 0.372 nm (Cs^+) (Supplementary Table 3). Correspondingly, when the sub-nanopore (geometric) mean-diameter was 0.82 nm, the observed $RSD_{0.82nm}$= 0.52 in the conductance was smaller than that observed for a sub-nanopore with a 0.3 nm diameter where $RSD_{0.30nm}$ = 1.56 (Fig. 2c and Supplementary Fig. 5).

Depending on the concentration, the conductance could be selective to the type of ion. For example, according to other work, the K^+-cation was supposed to be larger than sodium ($K^+(0.298\,nm) > Na^+(0.234\,nm)$) (Supplementary Table 3) and correspondingly, the K^+ conductance was observed (sometimes) to be larger than the Na^+ conductance in the same sub-nanopore, i.e. $g_{K^+} > g_{Na^+}$ (Fig. 2d and Supplementary Fig. 5). For example, in a sub-nanopore with a 0.4 nm mean diameter, $g_{K^+} = 0.66(1.39)\,nS > g_{Na^+} = 0.44(0.80)\,nS$ with $RSD_{0.4nm}(K)$ = 0.20(0.13) and $RSD_{0.4nm}(Na)$ = 0.38(0.18) for concentrations of 250(500) mM, respectively. However, for a concentration of 125 mM, $g_{K^+} = 0.28\,nS < g_{Na^+} = 0.31\,nS$, but with $RSD_{0.4nm}(K)$ = 0.47 and $RSD_{0.4nm}(Na)$ = 0.60. Based on the RSD, the assertion that $g_{K^+} > g_{Na^+}$ for concentrations > 250 mM survived the scatter in the conductance data acquired from the same pore. These data were also in-line with prior work based on MD that attributed the higher potassium conductance to the lower cost of constraining a hydrated K^+ inside the pore compared with the smaller hydrated Na^+. Potassium ions were supposed to allow for greater distortion of the hydration shell, and so the larger ion should permeate a smaller pore more easily.[8,9] Consistent with this premise, since Li^+ and Mg^{2+} were supposed to be more strongly hydrated[2,10] with diameters such that $K^+(0.298\,nm) > Li^+(0.188\,nm) > Mg^{2+}(0.144\,nm)$, it makes sense then that $g_{K^+} > g_{Li^+} > g_{Mg^{2+}}$, as was often observed (despite the low activity for Mg^{2+}), but not for every pore diameter. And since it was observed that $g_{Na^+} \geq g_{K^+}$ for more dilute concentrations (125 mM), which coincidentally corresponds to less than one ion in the pore volume, the data indicated that interactions between ions might play a role in selectivity for higher concentrations.

SUPPLEMENTARY TABLE 3. A tabulation of the ionic diameters. The diameters reported in this work, inferred from either the sub-nanopore conductivity or noise measurements, are compared with data reported in supplementary references 2-7. The hydrated diameters depend on the coordination of the water molecules.

ion	ionic diameter (This work) (nm)	ionic diameter (nm)	hydrated ionic diameter (nm)
H_3O^+	0.16 ± 0.11	0.230;[3]<br>0.266;[6]	0.560;[3]<br>0.392;[4]<br>0.200;[7]<br>0.550;[6]
Mg^{2+}	0.12 ± 0.11	0.144;[3]<br>0.129-0.138;[6]<br>0.144;[5]	0.856;[3]<br>0.600;[4]<br>0.418;[6]
Li^+	0.12 ± 0.11	0.188;[3]<br>0.152;[3]<br>0.118;[5]<br>0.132;[6]	0.764;[3]<br>0.120, 0.148;[2]<br>0.316;[4]<br>0.416;[6]
Na^+	0.21 ± 0.11	0.234;[3]<br>0.204;[4]<br>0.204;[5]<br>0.186;[6]	0.716;[3]<br>0.204, 0.214, 0.328, 0.346;[2]<br>0.402;[4]<br>0.470;[6]
K^+	0.23 ± 0.11	0.298;[3]<br>0.256;[4]<br>0.302;[5]<br>0.274;[6]	0.662,[3]<br>0.276, 0.292;[2]<br>0.402;[4]<br>0.558;[6]
Cs^+		0.372;[3]<br>0.348;[5]<br>0.342;[6]	0.658;[3]<br>0.346;[2]<br>0.626;[6]
Cl^-		0.328[3]<br>0.352;[6]	0.664[3]<br>0.636;[6]

SUPPLEMENTARY FIGURE 8.

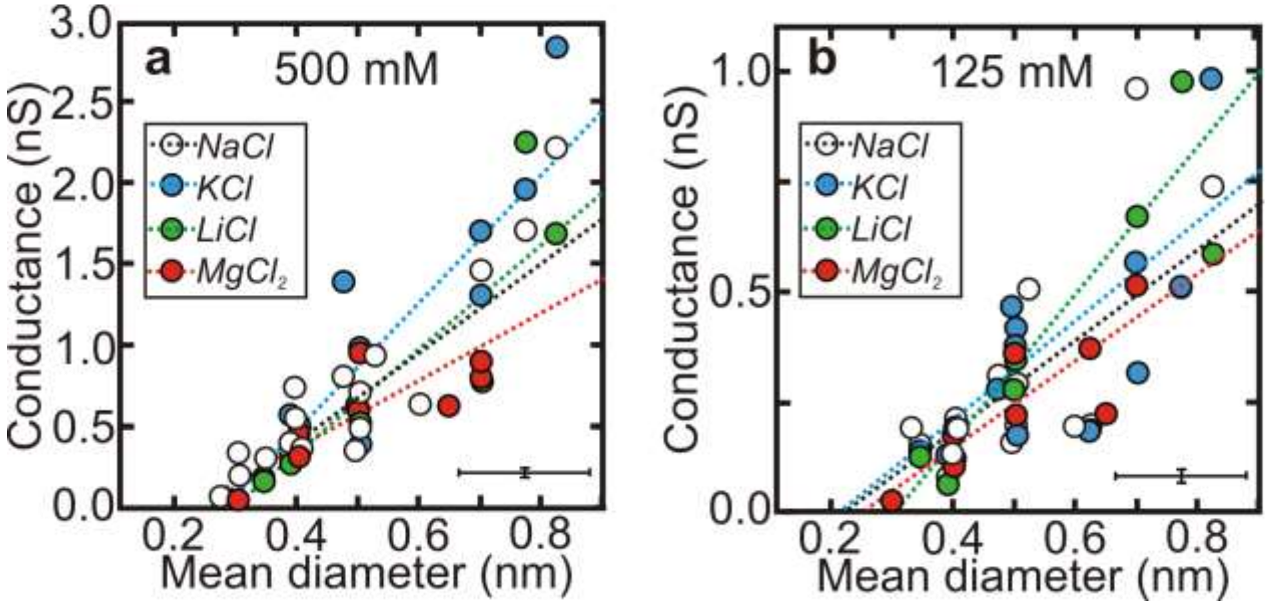

SUPPLEMENTARY FIGURE 8. The collapse of electrolytic transport through a sub-nanopore. (a,b) The dependences of the conductance on the (geometric) mean-diameter of sub-nanopores are shown for four different electrolytes (*NaCl*, *KCl*, *LiCl* and *$MgCl_2$*) for concentrations of 500 and 125 mM, respectively. The best-fit (dotted) lines extrapolate to zero conductance near mean-diameters of: 0.23 ± 0.11 nm for Na^+; 0.27 ± 0.11 nm for K^+; 0.30 ± 0.11 nm for Li^+; and 0.23 ± 0.11nm for Mg^+ at 500 mM; and 0.23 ± 0.11 nm for Na^+; 0.23 ± 0.11 nm for K^+; 0.31 ± 0.11 nm for Li^+; and 0.26 ± 0.11nm for Mg^+ at 125 mM concentration.

SUPPLEMENTARY NOTE #3: Conductance extrapolations for thick membranes.

If the membrane was considered to be thick compared to the diameter (and the surface charge was ignored for concentrations > 100 mM), then the point contact conductance (corrected for the finite thickness of the membrane) adds in series with the pore conductance, which scales like: $g = (\pi d_1 d / 4t) \times \sigma$, for a bi-conical pore through a membrane t thick, where d and d_1 represent the diameters at the waist and orifice, respectively, and so $g = \sigma / [(4t / \pi d_1 d) + (1/d)]$. Thus, the relative contributions of the access and pore resistance to the total depend on the cone-angle defined by the ratio or d_1/t. From this analysis, for the range of cone-angles relevant to this work, the pore resistance predominated and so the conductance was supposed to scale like: $g \sim d^2 \times \sigma$. Moreover, since the diffusivity is inversely proportional to the viscosity, which is inversely proportional to the diameter,[11,12] the conductance could collapse even faster—cubically with the diameter. Thus, allowing for a thick membrane, the power-laws governing the dependence on diameter were extracted from fits over the entire range of the data revealing that the conductance scaled like: $g \sim d^{\alpha}$ with α = 2.79, 2.38, 2.93 and 3.079 for *NaCl*, *KCl*, *LiCl* and

$MgCl_2$, respectively, but with marginally inferior R-squared: i.e. R^2 = 0.86, 0.85, 0.94 and 0.85, respectively. These fits on average intercept the conductance noise floor (10 pS) at d_M = 0.19 ± 0.11 nm. So, regardless of the scheme used for extrapolation, the conductance apparently vanished when the pore diameter shrunk below d_M = 0.24 ± 0.11 nm, commensurate with other estimates of the (de-hydrated) cation diameters.

SUPPLEMENTARY TABLE 4. Typical pH measurements of the electrolytic solutions. The pH of the de-ionized water (DI) was not measured directly, but inferred from in-line measurements of the resistivity. The slightly acidic pH generally observed in the electrolytes was attributed to CO_2 dissolved in the DI water.

electrolyte	pH (@ 125 mM/250 mM/500 mM/1 M/2 M)@ 22±1 °C
DI (18.2 MΩ·cm)	7 (inferred from in-line conductivity measurements)
DI (adulterated with *KCl*)	5.9
NaCl	5.7/5.7/5.7/5.7/5.6
KCl	5.7/5.8/5.7/–/–
LiCl	5.9/6.2/6.3/–/–
CsCl	5.7/5.5/5.4/–/–
$MgCl_2$	6.4/6.7/7.0/–/–

SUPPLEMENTARY NOTE #4: Proton conductance measurements.

Due to its small size, it was speculated that a proton could permeate through a sub-nanopore via a Grotthaus-like mechanism. According to MD simulations, a proton can tunnel across a series of short, hydrogen bonds, so-called "water wires", between hydronium ions and water molecules in the pore.[13] The proton motion is supposed to be associated with extreme, highly-correlated excursions across the network of hydrogen bonds. The electrolyte solutions were all slightly acidic pH 6 (Methods and Supplementary Table 4), but since the proton concentration at pH 6 was miniscule in comparison to the metal and chlorine ions ($[H^+]$ = 1 μM), the conductance vanished in the smallest sub-nanopores (Fig. 2f). On the other hand, it was reasoned that in the absence of other electrolyte, at a lower pH, the proton concentration $[H^+]$ would increase and the conductance along with it, even if the pore diameter was smaller than a water molecule.[13] What was critical to this argument is the idea that, when a hydrated excess proton encountered a hydrophobic volume devoid of water (near the pore waist), it could create its own water wire and fill the volume—wetting the dry space if only intermittently.

To test this idea, after wetting the pore in 250 mM *NaCl* electrolyte, the electrolyte was flushed, the pore stood in 18.2 MΩ–cm de-ionized water for 1 da, and then pure, concentrated hydrochloric acid (*HCl*) was introduced into the pore to decrease the pH to 1 ($[H^+]$ = 100 mM). Concentrated *HCl* was chosen because it supposedly does not etch silicon nitride with low oxide content.[14] Typically, the time-averaged conductance increased in concentrated *HCl* regardless of the pore diameter (Supplementary Fig. 9a). This increase could be attributed, at least in part, to the increased diffusivity of protons over metal cations. In bulk, the diffusivity of protons (D_{H+}) is supposed to be much larger (D_{H+} ~ 9.38 nm^2/ns) than that attributed to either metal cations (e.g. D_{Na+} = 1.33 nm^2/ns) or chlorine (D_{Cl-}= 0.79 nm^2/ns) anions. However, unlike the current measured in pH 6 electrolyte (Supplementary Fig. 9b; red traces), the current traces acquired from sub-nanopores in concentrated *HCl* fluctuated erratically at high voltage (Supplementary Fig. 9b; blue traces)—switching from a miniscule value to > 4 nS over a limited 1-2 s duration for both voltage polarities at 0.6V, for example. The transient peak current represented a phenomenal increase in the proton diffusivity: i.e. D_H^+ >13 nm^2/ns.

Moreover, unlike the 1/*f* noise observed in the same pore at 250 mM *NaCl* (Supplementary Fig. 9c; red trace), a typical power spectrum associated with the switching noise detected in *HCl* at pH 1 was better fit to a Lorentzian $S(f) = 2\tau/[1+(2\pi\tau f)^2]$, where f is the frequency in the range $0.01 < f < 10$, with a single life-time τ = 0.2 ± 0.1 s for a 0.27 nm-mean-diameter pore (whereas τ = 1.6 ± 0.1 s for a 0.8 nm-mean-diameter). Switching noise similar to this has been observed before in the electrolytic conduction through nanopores and attributed to fluctuations in the surface charge due to protonation and de-protonation.[15-17] Doubtless surface charge fluctuations could affect the conductance in a sub-nanopore as well, but the extended life-time and amplitude were inconsistent with the kinetics measured in nanopores in silicon nitride (τ= 5.6 μs).[15] So, taken altogether, the pulsatile current along with the Lorentzian current noise power spectrum, were more in-line with extreme, highly-correlated but short-lived proton excursions across the network of hydrogen bonds in the pore.

Since the data was acquired from pores larger than a water molecule (0.28 nm), a linear extrapolation of the dependence of the time-averaged proton conductance measured at pH 1 on the average sub-nanopore diameter was still used to inform on the electrolytic transport. This extrapolation indicated a vanishing conductance at $d_{H^+} = 0.16 \pm 0.11\, nm$ (Supplementary Fig. 9d; black dotted lines and circles), which was smaller than observed for Na^+ ions in the same pores

before exposure to concentrated *HCl*, i.e. $d_{Na^+} = 0.26 \pm 0.11\,nm$ (Supplementary Fig. 9d; light gray dotted lines and open circles), but within the error.

To determine if the pore topography was etched by the acid, after measuring the conductance, the acid was flushed with (18.2 MΩ–cm) de-ionized water and let stand for 1 da, and then immersed again in 250mM *NaCl* electrolyte and the conductance re-measured (Supplementary Fig. 9d; dark gray lines and gray circles). This extrapolation of the pore conductance likewise vanished at a mean pore diameter of $d_{Na^+} = 0.23 \pm 0.11\,nm$, which was consistent with the idea that the pore topography was immutable, but this interpretation was equivocal. If the Na^+ hydration shell was distorted when cations were forced through a sub-nanopore, then extrapolations incorporating all the data even that acquired from the larger, 0.9 to 1 nm-diameter pores may not provide a reliable estimate of the de-hydrated cation or proton size. Accordingly, extrapolations over the range in data acquired from mean-diameters between 0.25 nm to 0.7 nm, produced the same estimate for the Na^+ cation: i.e. $d_{Na^+} = 0.26 \pm 0.11\,nm$ before exposure to *HCl*, but indicated that $d_{H^+} = d_{Na^+} = 0 \pm 0.11\,nm$ afterward, which implied to the contrary that the pore topography may have been etched by *HCl*, enlarging it by about 0.2 nm.

The conductance in the sub-nanopores measured in pH 6, 250 mM *NaCl* electrolyte also improved by as much as 50% (Supplementary Fig. 9e; gray circles) after exposure to concentrated *HCl* over the conductance measured through a pristine pore in 250 mM *NaCl* (Supplementary Fig. 9e; open circles), which could be consistent with either an enlarged pore or instead reflect a reduction in the negative surface charge allowing for an increase in Cl^- ion flux. The possibility that the surface charge in the pore had changed after exposure to concentrated *HCl* was tested by imposing a gradient across the membrane (i.e. *NaCl* on the *trans*-side of the membrane was diluted to 1mM with 250mM *NaCl* on the *cis*-side) and measuring the conductance, but the asymmetry in the current-voltage characteristics with positive/negative bias deteriorated only marginally (Supplementary Fig. 9f), which was interpreted to reflect hardly any change in the surface charge. However, repeating the same procedure several times revealed that, whereas the *NaCl* conductance increased after the initial exposure to *HCl*, subsequent exposures to the acid produced little change in the *NaCl* conductance (Supplementary Fig. 9e; red circles), which ostensibly seemed to contradict the idea that the pore was being etched. Thus, the extrapolations over the entire range of sub-nanopore diameters, along with the reproducibility of the *NaCl* conductance measurements after repeated exposure to *HCl* seemed to indicate that the

acid had little effect on the pore topography, whereas extrapolations over a limited data set including only average diameters ≤ 0.7 nm indicated that the pore was etched and so, the possibility that acid etched the pore could not be discounted unequivocally.

SUPPLEMENTARY FIGURE 9.

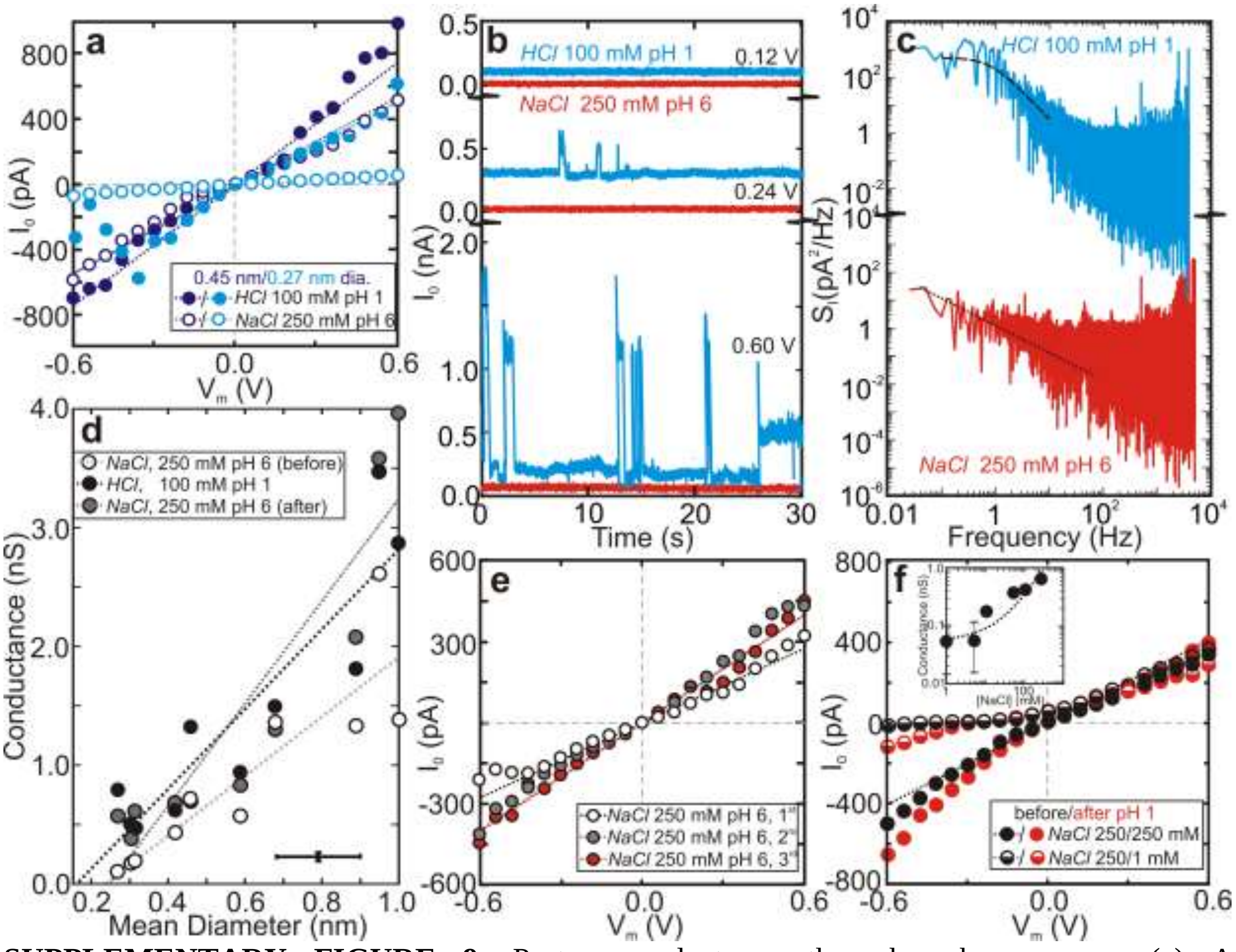

SUPPLEMENTARY FIGURE 9. Proton conductance through sub-nanopores. (a) A juxtaposition of the measured current-voltage characteristics is shown acquired from pores with (0.30 nm × 0.60 nm→) 0.45 nm and (0.25 nm × 0.30 nm→) 0.27 nm mean-diameter at the waist in 250 mM *NaCl* electrolyte (pH 6) and in 100 mM *HCl* (pH 1). (The dotted lines through the data represent Finite Element Simulations (FESs) of the characteristics.) (b) Current traces through a sub-nanopore with a mean-diameter of 0.27 nm acquired in *HCl* are shown at three voltages, 0.12, 0.24 and 0.60 V. Switching noise was observed, especially at high voltage when the sub-nanopore was immersed in *HCl* (blue), but not in 250 mM *NaCl* (red). (c) A comparison between the noise Power Spectral Density (PSD) acquired from a sub-nanopore with a mean-diameter of 0.27 nm at the waist in *HCl* (blue) and in *NaCl* a (red) at 240 mV. The two spectra are offset for clarity. In concentrated acid, the PSD can be fit to a Lorentzian (dashed line) at low frequency with $\tau = 0.2$ sec. On the other hand, in *NaCl*, the data is well represented by a fit to $1/f$ (dotted line). (d) The dependences of the conductance on the geometric mean-diameter of sub-nanopores are shown in *HCl* (black circles) and in 250 mM *NaCl*, prior to exposure of the

pore to *HCl* (open circles) and after (gray circles). The best-fit lines for 250 mM *NaCl* before/after the pores were exposed to *HCl* extrapolated to zero conductance near a mean-diameter of 0.23 nm/0.26 nm, respectively. For comparison, the conductance measured in *HCl* extrapolated to 0.16 nm. (e) Like (a), but comparing data acquired from the same pore with a mean diameter of (0.39 nm × 0.45 nm→) 0.42 nm at the waist in 250 mM *NaCl* electrolyte is shown before and after repeated exposures to *HCl*. (f) The conductance of a sub-nanopore with a mean-diameter of (0.65 nm × 0.70 nm→) 0.67 nm is shown, which was measured in 250 mM *NaCl* on both the *cis*- and *trans*-sides of the membrane (open circle), and then with only 1 mM *NaCl* on the *trans*-side (half-filled circles) to illustrate selectivity to (Na^+) cations before and after exposure to concentrated *HCl*. The dotted lines represent FESs that were used to match the data. The asymmetric conductance with bias polarity indicates that > 97 % of the current was carried by Na^+ both before and after exposure to the acid. Inset: The concentration dependence of the conductance in the same pore is shown. The dotted-line represents a match to the data assuming a lower bound on the surface charge of about 0.11 e^-/nm^2.

SUPPLEMENTARY FIGURE 10.

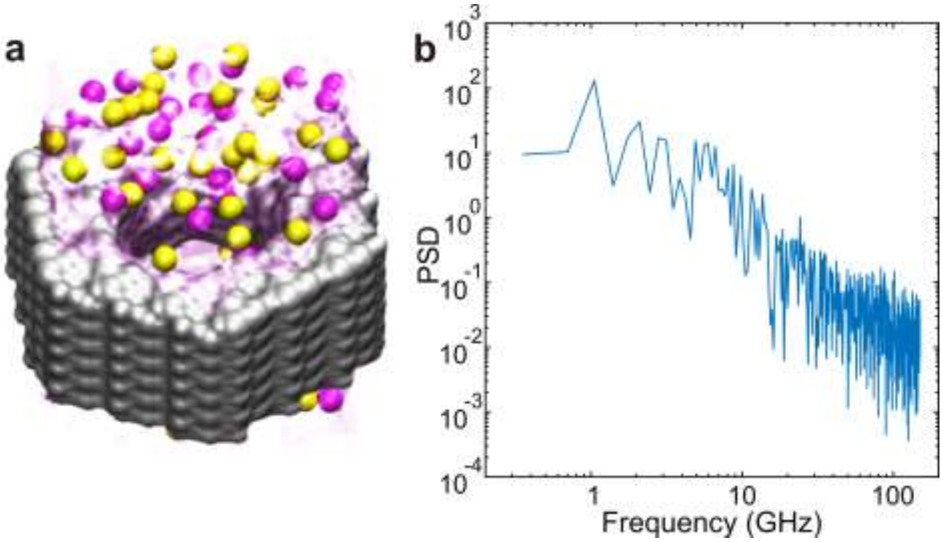

SUPPLEMENTARY FIGURE 10. Molecular Dynamics (MD) simulations of a sub-nanopore. MD offers an atomistic perspective of the current, but it is difficult to make contact with experiment using MD because the simulations are incommensurate with the limited bandwidth and/or low electric fields and electric field distribution that are inherent in the measurements. This figure illustrates the essential features of a MD simulation. (a) The figure shows the topography of a 1 nm diameter pore through a 5 nm thick α–Si_3N_4 membrane. The pore was immersed in a 1M *NaCl* solution (water is not shown) with a 3 V transmembrane potential applied (b) The noise power spectral density is shown. The pore current varied from 10-20 nA with a standard deviation of 7-8 nA, which corresponded poorly with the empirical data (shown in Figs 4a-c) because of the simulation time-frame; i.e the base-line frequency (< 1 nsec) is just too short. These simulations are in stark contrast with the empirical data acquired by analyzing the current over 30 sec intervals. In particular, the resulting $1/f$ noise data was acquired in the frequency range from 0.05 to 1 kHz—not GHz. To reconcile the simulations with the experiments, it would be necessary to simulate for > 1 sec (which is currently impractical), and then impose an appropriate average. The basic problem is that classical MD simulations scale like the number of atoms as $\sim N^2$ and so are usually restricted to a few nanoseconds for economy

(i.e. simulations of 50,000 atoms are accomplished in about 1 nsec with time-steps ranging from 0.4 to 2 fs).

SUPPLEMENTARY FIGURE 11.

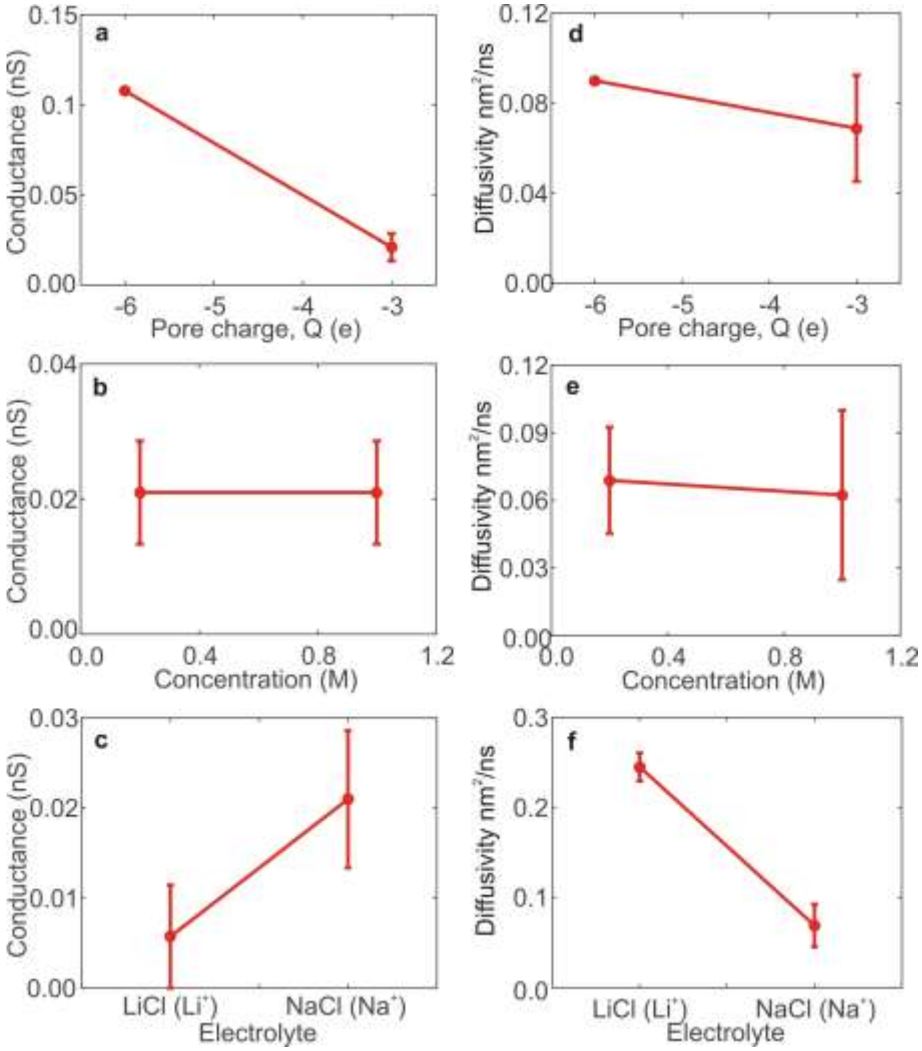

SUPPLEMENTARY FIGURE 11. Cation conductance and diffusivity extracted from Molecular Dynamics (MD) simulations. (The total simulation time was 52 ns for each case and since only the counter-ions permeate through the sub-nanopores, only the cation conductance and diffusivities are shown.) (a,d)The conductance and diffusivity, respectively, through a pore with a diameter of 0.30 nm for two different total pore charges of $Q = -3.0e$ and $-6.0e$ are shown. A pore diameter with $d = 0.3$ nm diameter at the waist (see Fig. 1f), immersed in 200 mM *NaCl*, was used for the simulation. (b,e) The conductance and diffusivity, respectively, are shown through a pore with a diameter of 0.30 nm for two different concentrations 200 mM and 1.0 M of *NaCl* electrolyte. The total pore charge is $Q = -3.0e$. (c,f) The conductance and diffusivity, respectively, through a pore with a diameter of 0.30 nm for two different electrolytes, *LiCl* and *NaCl*, are shown. Since only the cations (Li^+, Na^+) permeated through the sub-nanopore, actually the plot compares the values of Li^+ and Na^+. In the simulation, the salt concentration was 200 mM and the total pore charge was $Q = -3.0e$.

SUPPLEMENTARY FIGURE 12.

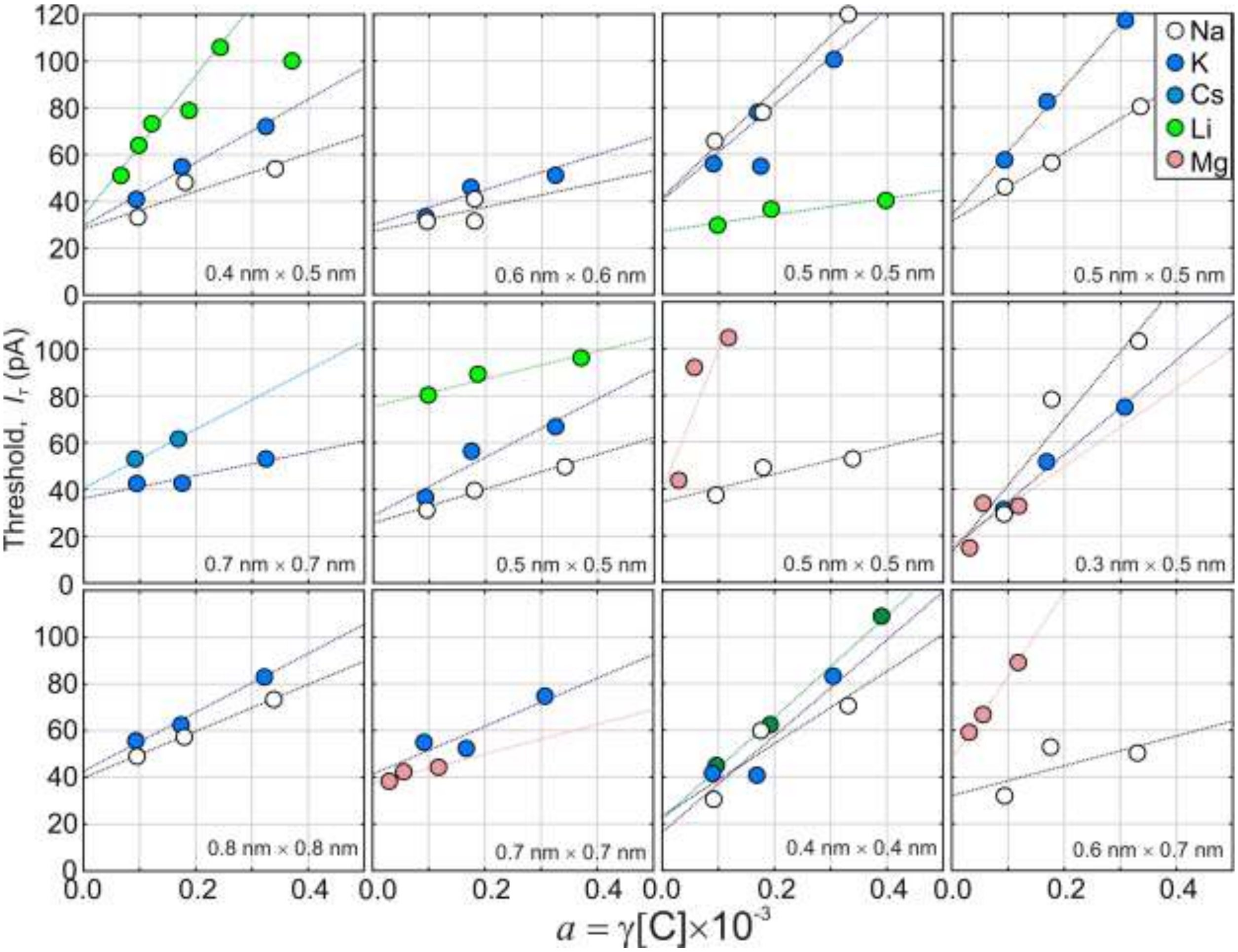

SUPPLEMENTARY FIGURE 12. Typical data illustrating the dependence of the noise threshold, I_T, on the electrolytic activity for different sub-nanopore cross-sections at the waist. Data acquired in five different electrolytes (*NaCl*, *KCl*, *LiCl*, *$MgCl_2$* and *CsCl*) are shown. The best-fit lines extrapolate to I_{T0} at zero activity (or infinite dilution). The cross-sections of the sub-nanopores at the waist (lower right) were estimated through multi-slice simulations of actual TEM images.

SUPPLEMENTARY FIGURE 13.

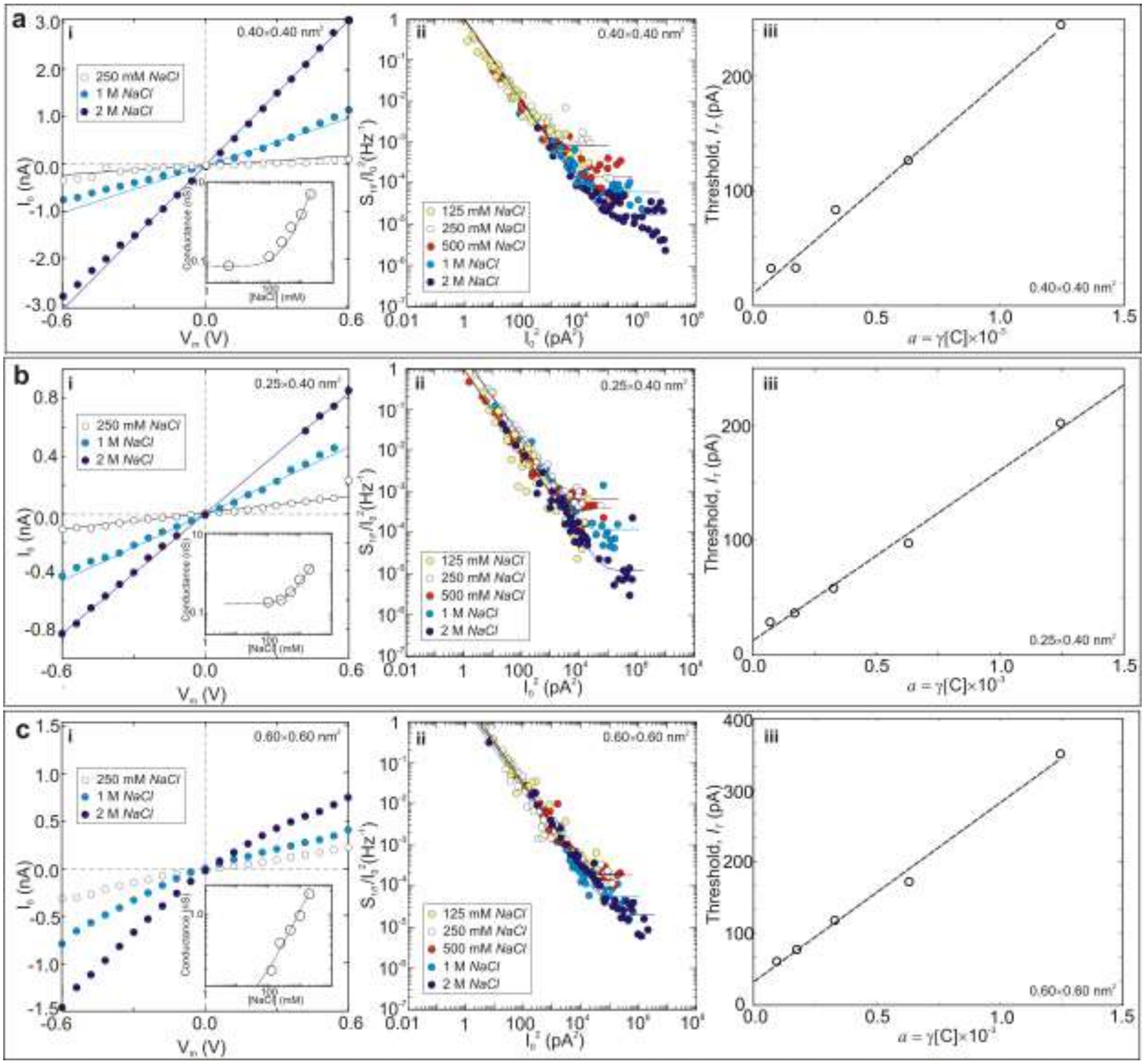

FIGURE 13. Correlated current noise in sub-nanopores. (a-c; i) A juxtaposition of the measured current-voltage characteristics are shown acquired from pores with (geometric) mean-diameters ranging from (0.40 nm × 0.40 nm→) 0.40 nm to (0.25 nm × 0.40 nm) →) 0.32 nm to (0.60 nm × 0.60 nm→) 0.60 nm, respectively, using different concentrations of *NaCl* electrolyte ranging from 125 mM to 2 M, as indicated. The cross-sections of the sub-nanopores at the waist were estimated through multi-slice simulations of transmission electron micrographs. (a-c; Insets) The conductances are shown as a function of *NaCl* concentration for the same pores. The dotted-line represents a match/fit to the data assuming a negative surface charge in the pores. (a-c; ii) The (pink) noise Power Spectral Density (PSD) intercept at 1Hz ($S_{1/f}$), normalized by the square of the open pore current, I_0^2, measured in *NaCl* electrolyte, ranging in concentration from 125 mM to 2 M, is plotted as a function of I_0^2 for the respective sub-nanopores. The normalized noise power for $I_0 < 10$ pA was generally consistent with noise resulting from uncorrelated current fluctuations. However, beyond the threshold current defined by I_T, S_I / I_0^2 is independent of the

current indicating correlations between fluctuations, except for 125 mM concentration. The solid lines represent fits to the data. (a-c; iii) Data illustrating the dependence of the noise threshold, I_T, on the electrolytic activity for the same sub-nanopores is shown. The best-fit lines extrapolate to zero activity (or infinite dilution) at I_{T0} = 10.3, 11.7 and 33.3 pA, respectively.

SUPPLEMENTARY FIGURE 14.

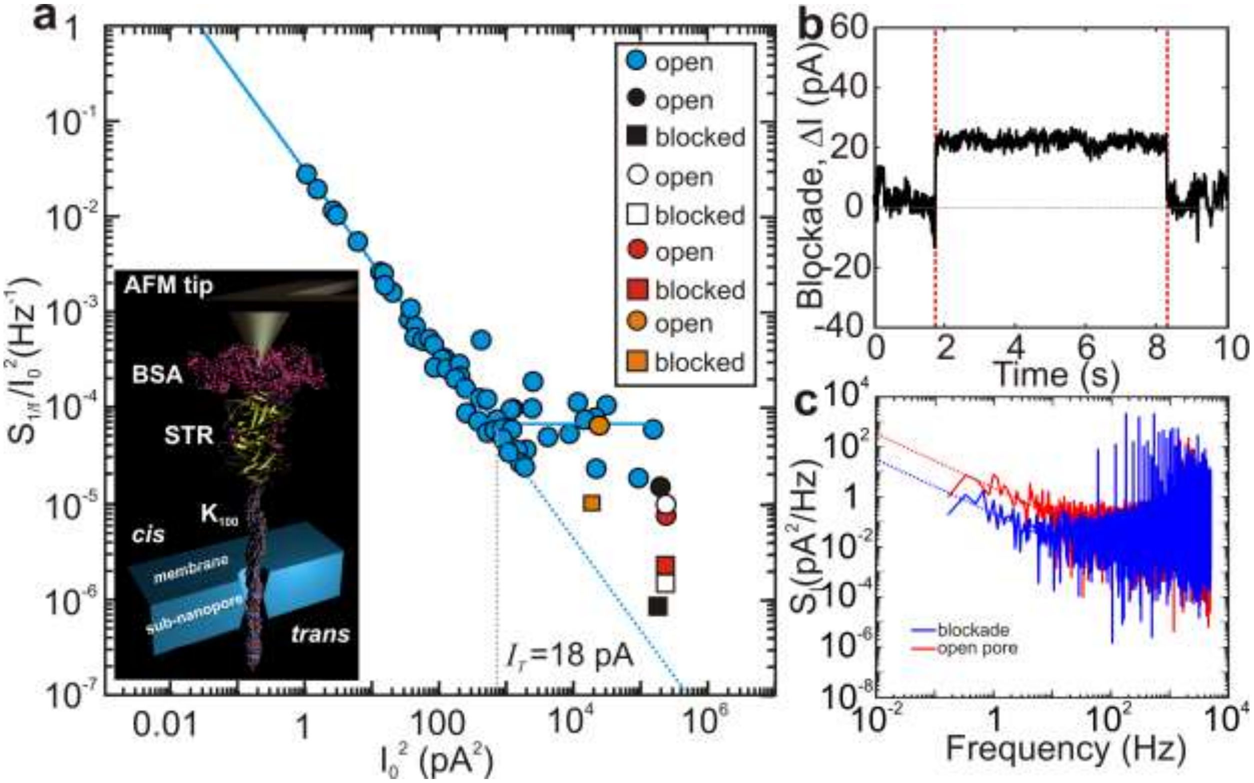

SUPPLEMENTARY FIGURE 14. Current noise observed during current blockades of a sub-nanopore by a K_{100} homopolymer protein. A denatured homopolymer protein, poly-L-lysine (K_{100}), tethered to the tip of an AFM cantilever, was forced into a sub-nanopore with a mean-diameter of (0.40 × 0.50 nm^2 →) 0.45 nm at the waist. With K_{100} blockading the electrolytic current through the sub-nanopore, the unoccluded volume was reduced and so correlations in the ion transport were disrupted, which diminished the noise. This K_{100} homopolymer was chosen because the van der Waals volume (in equilibrium) of a lysine residue is about 0.1713 nm^3 (0.76 nm wide × 0.38 nm long), which should therefore overfill the sub-nanopore waist even if it is stretched.[16] It was especially interesting that, even though the terminal amino group in the residue was charged, the three methylene groups comprising the side-chain in lysine had significant hydrophobic character. Thus, the side-chains were supposed to create a hydrophobic volume within the waist. Finally, lysine is positively charged at neutral pH and the pKa of the lysine side chain is > 10 so that it was supposed to remain protonated for pH < 7. Negatively charged Sodium Dodecyl Sulfate (SDS), which was used to stabilize the denatured protein, should therefore readily bind to K_{100} forming a protein-SDS aggregate consisting of a shell along the length of the protein backbone that facilitated the translocation by an electric force field.[18] (a) The integrated (pink) noise Power Spectral Density (PSD) in the range 0.01 Hz to 10 Hz (S_I), normalized by the square of the open pore current, I_0^2, measured in 250 mM *NaCl* electrolyte (blue) is plotted as a function of I_0^2 for a sub-nanopore with a mean diameter of 0.45 nm at the waist. The normalized noise power for I_0 < 10 pA was generally consistent with noise resulting

from uncorrelated current fluctuations until a threshold current I_t (delineated by the gray dotted line at $I_T = 17.6\,pA$). The blue dotted line offers a linear extrapolation of S_I / I_0^2 with I_0^2. Beyond the threshold, S_I / I_0^2 was independent of the current indicating correlations between fluctuations, except when there was a current blockade (white, red, black square symbols). The data represented by the blue and black symbols was acquired from the same pore whereas the data represented by the orange/white/red symbols were obtained from another, practically identical pore. Inset: Cutaway of the schematic showing a biotinylated K_{100} homopolymer, tethered to the tip of an AFM cantilever through a bond to STReptavidin (STR), translocating through the sub-nanopore. (b) A current trace of a blockade, ΔI, associated with a single K_{100} homopolymer, tethered to an AFM cantilever, as it was impelled through the same pore under the same conditions in (a). To measure a blockade current, a transmembrane voltage of 0.7 V was applied to the pore resulting in an open pore current of about $I_0 = 159\,pA$ and then a single K_{100} homopolymer was threaded through the pore and retracted from it at a constant velocity of 4 nm/s, which translated to a molecule trapped for 10-20 s in the pore. This bias voltage was chosen to minimize the fractional change in the current due to the blockade. Since the volume of the lysine residue was about 0.1713 nm^3, the corresponding fractional change in volume associated with twenty-six AAs spanning the entire membrane would be about: $\Delta V_{26AA} / V_{pore} = 4.5 / 29.7 = 0.15 \propto \Delta I / I_0$ or $\Delta I \sim 24\,pA$ for $I_0 = 159\,pA$, assuming a pore volume of 29.7 nm^3 associated with a bi-conical pore topography with a 15° cone angle that has a 0.45 nm diameter at the waist, which coincidently was comparable to the empirical value shown in Supplementary Fig. 14b. Apparently, the introduction of the protein-SDS aggregate reduced the unoccluded volume enough to disrupt the correlations in the ion motion. The current noise in the blockade was suppressed (Supplementary Figs. 14b,c). The Coefficient of Variation, $(CV \equiv \sigma_{\Delta I} / \Delta I)$ which was used to gauge the fluctuation amplitude associated with time-dependent fluctuations, changed from $CV = 0.26, \Delta I_{rms} = 4.1\,pA$ to a third of that value: $CV = 0.082, \Delta I_{rms} = 1.7\,pA$ during the blockade. (c) The PSD acquired from the same pore with (blue) and without (red) a homopolymer blockade is shown. Whereas the normalized 1/*f*-PSD associated with the open pore current was generally consistent with the excess noise associated with the correlated ion motion above threshold (Supplementary Fig. 14a; orange, red, white and black circles), the PSD of the blockade current was not—instead, it was nearly collinear (Supplementary Fig. 14a; blue dotted line) with the normalized noise measured at much lower currents in the same pore (Supplementary Fig. 14a; orange, red, white and black squares). From this data, it was deduced that the correlation between Na^+ ions must have been disrupted during transport through the unoccluded volume.

SUPPLEMENTARY REFERENCES.

1. Levin, E.V., Burns, M. M. & Golovchenko, J. A. Nanoscale dynamics of Joule heating and bubble nucleation in a solid-state nanopore. *Phys. Rev. E* **93**, 013124 (2016).
2. Mähler, J. & Persson, I. A study of the hydration of alkali metal ions in aqueous solution. *Inorg. Chem.* **51**, 425-438 (2012).
3. Volkov, A.G., Paula, S. & Deamer, D. W. Two mechanisms of permeation of small neutral molecules and hydrated ions across phospholipid bilayers. *Bioelectrochem. Bioenergetics* **42**, 153-160 (1997).
4. Kiriukhin, M.Y. & Collins K.D. Dynamic hydration numbers for biologically important ions, *Biophys. Chem.* **99**, 155–168 (2002).

5. Shannon, R.D. Revised effective ionic radii and systematic studies of interactomic distances in halides and chalcogenides. *Acta Crystallogr.* **A32**, 751-767 (1976).
6. Marcus, Y. Ionic Radii in Aqueous Solutions, *Chem. Rev.* **88**, 1475-1498 (1988).
7. Marcus, Y. Volumes of aqueous hydrogen and hydroxide ions at 0 to 200 °C, *J. Chem. Phys.* **137**, 154501 (2012).
8. Li, H., Francisco, J. S. & Zeng, X.C. Unraveling the mechanism of selective ion transport in hydrophobic subnanometer channels. *Proc. Nat. Acad. Sci. U.S.A.* **112**, 10851-10856 (2015).
9. Carrillo-Tripp, M., Saint-Martin, H., Ortega-Blake, I. Minimalist molecular model for nanopore selectivity. *Phys. Rev. Lett.* **93**, 1168104–1168111 (2004).
10. Smith, D.W. Ionic Hydration Enthalpies. *J. Chem. Educ.* **54**, 540−542 (1977).
11. Ortiz-Young, D., Chiu, H. C., Kim, S., Voïtchovsky, K. & Riedo, E. The interplay between apparent viscosity and wettability in nanoconfined water. *Nature Comm.* **4**, 2482 (2013).
12. Gao, J., Szoszkiewicz, R., Landman, U. & Riedo, E. Structured and viscous water in sub-nanometer gaps. *Phys. Rev. B* **75**, 115415-1-6 (2007).
13. Peng, Y., Swanson, J. M., Kang, S-G., Zhou, R. & Voth, G. A. Hydrated Excess Protons Can Create Their Own Water Wires. *J. Phys. Chem. B.* **119**, 9212-9218 (2015).
14. Bermudex, V.M. Wet-chemical treatment of Si_3N_4 surfaces studied using infrared attenuated total reflection spectroscopy, *J. Electrochemical Soc.* **152**, F31-F36 (2005).
15. Hoogerheide, D.P., Garaj, S. & Golovchenko, J. A. Probing surface charge fluctuations with solid-state nanopores. *Phys. Rev. Lett.* **102**, 256804 (2009).
16. Jain, T., Rasera, B. C., Guerrero, R. J. S., Boutilier, M. S. H., O'Hern, S. C., Idrobo, J-C. & Karnik, R. Heterogeneous sub-continuum ionic transport in statistically isolated graphene nanopores. *Nat. Nanotech.* **10**, 1053-1058 (2015).
17. Bezrukov, S.M. & Kasianowicz, J.J. Current noise reveals protonation kinetics and number of ionizable sites in an open protein ion channel. *Phys. Rev. Lett.* **70**, 2352–2355 (1993).
18. Dong, Z., Kennedy, E., Hokmabadi, M. & Timp, G. Discriminating residue substitutions in a single protein molecule using a sub-nanopore. *ACS Nano* **11**, 5440-5452 (2017).
